# Oxidized High-Density Lipoprotein Induces Endothelial Fibrosis Promoting Hyperpermeability, Hypotension, and Increased Mortality

**DOI:** 10.3390/antiox11122469

**Published:** 2022-12-15

**Authors:** Macarena Rojas, Yolanda Prado, Pablo Tapia, Leandro J. Carreño, Claudio Cabello-Verrugio, Felipe Simon

**Affiliations:** 1 Laboratory of Integrative Physiopathology, Faculty of Life Science, Universidad Andres Bello, Santiago 8370186, Chile; 2 Millennium Institute on Immunology and Immunotherapy, Santiago 8331150, Chile; 3 Unidad de Paciente Crítico Adulto, Hospital Clínico La Florida, La Florida, Santiago 8242238, Chile; 4 Programa de Inmunología, Instituto de Ciencias Biomédicas, Facultad de Medicina, Universidad de Chile, Santiago 8380453, Chile; 5 Laboratory of Muscle Pathology, Fragility and Aging, Faculty of Life Science, Universidad Andres Bello, Santiago 8370186, Chile; 6 Center for the Development of Nanoscience and Nanotechnology (CEDENNA), Universidad de Santiago de Chile, Santiago 9170020, Chile; 7 Millennium Nucleus of Ion Channel-Associated Diseases (MiNICAD), Santiago 8380453, Chile

**Keywords:** oxHDL, oxidative stress, endothelium, fibrosis, blood pressure, mortality, hyperpermeability

## Abstract

During systemic inflammation, reactive oxygen species (ROS) are generated in the bloodstream, producing large amounts of oxidized HDL (oxHDL). OxHDL loses the vascular protective features of native HDL, acquiring detrimental actions. Systemic inflammation promotes endothelial fibrosis, characterized by adhesion protein downregulation and fibrotic-specific gene upregulation, disrupting endothelial monolayer integrity. Severe systemic inflammatory conditions, as found in critically ill patients in the intensive care unit (ICU), exhibit endothelial hyperpermeability, hypotension, and organ hypoperfusion, promoting organ dysfunction and increased mortality. Because endothelial fibrosis disturbs the endothelium, it is proposed that it is the cellular and molecular origin of endothelial hyperpermeability and the subsequent deleterious consequences. However, whether oxHDL is involved in this process is unknown. The aim of this study was to investigate the fibrotic effect of oxHDL on the endothelium, to elucidate the underlying molecular and cellular mechanism, and to determine its effects on vascular permeability, blood pressure, and mortality. The results showed that oxHDL induces endothelial fibrosis through the LOX-1/NOX-2/ROS/NF-κB pathway, TGF-β secretion, and ALK-5/Smad activation. OxHDL-treated rats showed endothelial hyperpermeability, hypotension, and an enhanced risk of death and mortality, which was prevented using an ALK-5 inhibitor and antioxidant diet consumption. Additionally, the ICU patients showed fibrotic endothelial cells, and the resuscitation fluid volume administered correlated with the plasma oxHDL levels associated with an elevated risk of death and mortality. We conclude that oxHDL generates endothelial fibrosis, impacting blood pressure regulation and survival.

## 1. Introduction

Lipoproteins circulate in the blood, completing broad functions in health and disease. The main lipoproteins include low-density lipoprotein (LDL) and high-density lipoprotein (HDL) [1]. Although LDL is related to several vascular diseases, HDL is accepted as a vascular protective factor that confers a defense against vascular diseases, such as coronary artery disease and atherosclerosis [2]. HDL exerts its protective effects by interacting with endothelial cells (ECs) to trigger the intracellular signaling that supports endothelial functions, including vascular permeability preservation and endothelium integrity [3,4,5,6].

Systemic inflammatory conditions cause an enhanced oxidative environment in the bloodstream mediated by reactive oxygen species (ROS) generation [7,8]. Therefore, during systemic inflammatory conditions, circulating ROS interact with plasma circulating macromolecules, promoting oxidative modification [9,10]. Thus, lipoproteins are highly oxidized in the bloodstream through modifications mediated through the reactions triggered by ROS and ROS-activated oxidative enzymes [11,12]. Importantly, HDL is more susceptible to oxidation than other lipoproteins; so, a large amount of oxidized HDL (oxHDL) is generated under oxidizing conditions [13]. Notably, oxHDL loses the protective features of HDL, acquiring detrimental actions on the endothelium and contributing to endothelial dysfunction [14,15]. It has been reported that oxHDL binds lectin-like oxidized low-density lipoprotein receptor 1 (LOX-1), promoting endothelial dysfunction through the activation of nuclear factor-κB (NF-κB) [16,17,18,19].

During systemic inflammatory conditions, ECs are converted into activated fibroblasts through the endothelial-to-mesenchymal transition (EndMT) process in response to several mediators of inflammation, including transforming growth factor-β1 (TGF-β1), TGF-β2, and endotoxin [20,21,22,23]. EndMT promotes a protein synthesis alteration that generates decreased endothelial adhesion proteins, such as VE۔cadherin, and increased fibroblast-specific proteins, such as α۔smooth muscle actin (α-SMA). Furthermore, the proteins that constitute the extracellular matrix (ECM), including fibronectin (FN), are also increased. Thus, a severe disruption of the cell-to-cell contact of the endothelial monolayer is produced, resulting in a fibroblast-like phenotype [20,23,24]. Currently, it is not known whether oxHDL promotes endothelial fibrosis and the underlying molecular mechanism.

Severe acute systemic inflammatory conditions, such as those found in critically ill patients admitted to the intensive care unit (ICU), are characterized by serious vascular alterations. ICU patients exhibit endothelial hyperpermeability associated with extensive edema formation, promoting decreased blood pressure, hypoperfusion to organs, and finally death [25,26,27]. Notably, vascular permeability is maintained by the expression of adhesion proteins, including VE۔cadherin, which bind ECs to each other, preventing vascular leaks [26,27,28]. Thus, because of endothelial fibrosis, decreased VE-cadherin expression, together with α-SMA and FN upregulation, mediates EC shape disruption, impairing the endothelial monolayer and leading to an increased endothelial permeability that ultimately promotes hypotension, hypoperfusion, organ dysfunction, and increased mortality [25,29,30].

However, whether oxHDL promotes endothelial fibrosis as a mechanism to induce endothelial hyperpermeability, blood pressure decrease, organ hypoperfusion, and increased mortality is not known. Therefore, the aim of this study was to investigate the fibrotic effect of oxHDL on the endothelium, elucidating the underlying molecular mechanism and determining its effects on vascular permeability, blood pressure, and mortality.

Our findings indicate that oxHDL generates endothelial fibrosis, impacting blood pressure control and survival. Notably, the inhibition of endothelial fibrosis appears to be a new approach to treating the adverse actions of increased levels of oxHDL during acute systemic inflammatory conditions.

## 2. Materials and Methods

### 2.1. Human Artery Endothelial Cell Culture and mRNA and Protein Expression Determination

Human aortic endothelial cells (HAEC, Lonza, Chicago, IL, USA) were cultured at 37 °C in a 5%:95% CO_2_:air atmosphere in an EGM-2 medium supplemented with 2% FBS, 2 mM glutamine, and 50 U/mL penicillin-streptomycin (Sigma-Aldrich, St Louis, MO, USA). On the day before the study, the FBS concentration was reduced to 1%. RT-qPCR experiments were performed to measure the VE-cadherin, α-SMA, FN, TGF-β1, and TGF-β2 mRNA levels in the ECs. Total RNA was extracted with Trizol according to the manufacturer’s protocol (Invitrogen, Carlsbad, CA, USA). DNAse I-treated RNA was used for reverse transcription using the Super Script II Kit (Invitrogen, USA). Equal amounts of RNA were used as templates in each reaction. Quantitative PCR was performed using the SYBR Green PCR Master Mix (AB Applied Biosystems, Waltham, MA, USA). Assays were run using a RotorGene instrument (Corbet Research, Mortlake, Australia). The data are presented as relative mRNA levels of the gene of interest normalized to the relative levels of 28S mRNA and normalized against the control condition. Flow cytometry analysis was performed to determine the expression changes of VE-cadherin, α-SMA, and FN, using the corresponding monoclonal antibodies (all from R&D Systems, Inc., Minneapolis, MN, USA). The labeled cells were then analyzed immediately by flow cytometry (BD FACS Fortessa, BD Biosciences, San Jose, CA, USA). Color compensation matrices were calculated for each staining combination within each experiment using a single-stained antibody. In all the analyses, the doublets and clusters were eliminated. A minimum of 10,000 events were analyzed. The secreted TGF-β1 and TGF-β2 were measured using ELISA kits in accordance with the manufacturer’s instructions (ThermoFisher, Waltham, MA, USA). Fluorescent immunocytochemistry was performed to detect VE-cadherin, α-SMA, and FN. The cells were washed twice with PBS and fixed. The cells were subsequently washed again and incubated with the first primary antibodies. Then, the cells were washed twice and incubated with the first secondary antibodies. The samples were mounted with ProLong Gold antifade mounting medium with DAPI (Invitrogen).

### 2.2. Oxidization of HDL

The native HDL (Sigma-Aldrich, USA) was oxidized as previously described [16,17], with modifications. Briefly, HDL at a final concentration of 0.5 mg/mL was incubated at 37 °C for 16 h in the presence of 50 μM CuSO_4_ in PBS. The reaction was stopped by storing the oxHDL at 4 °C to prevent further oxidation. The extent of lipoprotein oxidation was monitored by measuring the thiobarbituric acid reactive substances (TBARS) formation with the TBARS assay kit (Cayman Chemical Company, Ann Arbor, MI, USA), following the manufacturer’s instructions [31]. For the oxHDL, a total of 13.33 ± 2.23 μM MDA was obtained vs. 1.96 ± 0.43 μM from the native HDL, in a total of seven independent experiments (*p* = < 0.001). To chelate copper from the reaction, the oxHDL was incubated for 5 min with 100 mg/mL CHELEX-100 (Bio-Rad Laboratories Inc., Hercules, CA, USA), centrifuged at 4 °C for 1 min at 500×·*g*, and the pellet was discarded

### 2.3. Small Interfering RNA and Transfection

SiGENOME SMARTpool siRNA (four separated siRNAs per NOX-1, NOX-2, NOX-4, TGF-β1, and TGF-β2) were purchased from Dharmacon. In brief, the HAECs were plated overnight in a 24-well plate and then transfected with 5 nmol/L siRNA using the DharmaFECT 4 transfection reagent (Dharmacon Horizon, Lafayette, CO, USA) according to the manufacturer’s protocols in a serum-free medium for 6 h. After 48 of transfection, the experiments were performed.

### 2.4. Cell Death, Viability, Proliferation, and Migration

Cell death was performed using the trypan blue (0.05 % *w*/*v*) exclusion method. The cells were quantified by light microscopy using a hematocytometer chamber. In each dish, at least 300 cells were counted in 5 random fields. Cell viability was determined using the MTT colorimetric assay (ThermoFisher, USA), in which cell viability was quantified by the amount of MTT reduction. After the treatment was performed, the cells were co-incubated with anhydrous MTT for 4 h and then solubilized with an isopropanol/DMSO solution. The optical density value was measured at 540 nm. Cell proliferation was assessed by the BrdU incorporation method. After treatments, the cells were labeled with 20 μL/well BrdU labeling solution (ThermoFisher, USA). After incubation, the cells were washed three times with PBS, and a fixing solution was added. The absorbance was read at 450 nm. Cell migration was measured by wound-closure assay. The ECs were seeded until they reached a monolayer. Then, the cell cultures were scratched with a 10 μL sterile tip and washed to remove the detached cells and debris. After treatment, the cells were fixed with methanol for 2 min. Images of each wound were captured through a digital microscope, and the ratio of the wound closure percentage was determined

### 2.5. In Vitro Permeability Assay

The HAECs were cultured to full confluency for 24 h on a gelatin-coated Boyden chamber mounted inside the wells of 24-well plates (Transwells, Costar Transwell, 0.4 μm pore size, 12-mm diameter, Corning Inc., New York, NY, USA). The confluent monolayers were treated for 48 h with a vehicle, HDL (50 μg/mL), and oxHDL (0.5, 5, 50, 200 μg/mL). Moreover, the indicated experiments were treated for 1 h before and during treatment with Anti-LOX, κ-carr, DPI, Apo, GSH, and NAC. Then, FITC-dextran 40 kDa (0.5 mg/mL, Sigma-Aldrich, USA) was added to the chamber. After 90 min of incubation, a 50 μL sample was taken from the plate well, and the fluorescence was measured using a fluorescence plate reader (excitation 485 nm, emission 520 nm). Alternatively, the cells were transiently transfected with specific siRNA or non-targeting siRNA in separate plates. At twelve hours post-transfection, the cells were cultured to full confluency on gelatin-coated transwells.

### 2.6. Animals, Lipoprotein Administration, Blood Samples, and Parameter Recordings

Male Sprague Dawley (SD) rats weighing from 180 to 220 g were used. They were housed in cages with water and food ad libitum, a 12 h light/dark cycle, and a 25 ± 1 °C temperature. Before the experiments, no food was given for 12 h, while water was available all the time. All the experimental procedures with the rats were approved by the Commission of Bioethics and Biosafety of Universidad Andres Bello (N°002/2020) and followed the instructions from the Guide for the Care and Use of Laboratory Animals from the National Research Council and the American Association for Laboratory Animal Science. This research complies with the commonly accepted 3Rs. The rats were treated with saline solution (saline, 0.9% NaCl), HDL (0.4 mg/kg), and oxHDL (2 mg/kg), and a ratio of oxHDL/HDL (2/0.4 mg/kg) by intraperitoneal (I.P.) injection (200 µL) every 24 h for 4 weeks. The rat blood samples were collected in a sodium citrate blood collection Vacutainer^®^ tube through tail vein puncture or cardiac puncture after anesthetizing or when the death event occurred. For plasma extraction, the obtained blood was immediately centrifuged at 4000 rpm for 10 min at 4 °C to separate the plasma, which was immediately stored at −80 °C. Systolic pressure and heart rate were measured through the experiment. Moreover, survival analyses were performed throughout the experiment.

### 2.7. Systolic Blood Pressure and Heart Rate

Systolic blood pressure (P_S_) and heart rate (H_R_) were acquired with a physiological recording system and a pressure tail cuff for the noninvasive blood pressure recording system for rats (ML125/R), coupled with a MLT125/R pulse transducer (AD Instruments, Sydney, Australia). To perform the recordings of P_S_ and H_R_, the animals were recorded for 1 min several times to obtain reliable values. All the transducers were connected to a PowerLab^®^ 8/30 (AD Instruments), and the physiological variables were instantaneously displayed through Chart^®^ software (AD Instruments).

### 2.8. Primary Rat Mesenteric Artery Endothelial Cell (RMAEC) Isolation

Primary rat mesenteric artery endothelial cells (RMAECs) were isolated from the mesenteric artery. The mesenteric artery was occluded on its distal end and cannulated from its proximal end with a polyethylene tubing connected to a 21-gauge syringe. The mesentery was surgically removed and washed with sterile PBS. For the enzymatic isolation of the RMAECs, the mesenteric artery was slowly perfused in a culture hood for 5 min with 5 mL M-199 medium supplemented with 40 μL Pen/Strep (10,000 U/mL/10,000 μg/mL), 20 μL Fungizone (250 μg/mL), and 12.5 mg collagenase type II. The cell suspension was centrifuged at 3000 rpm for 7 min; the pellet was reconstituted in 3 mL M-199 medium supplemented with 8 mL/L of Pen/Strep (10,000 U/mL/10,000 μg/mL), 4 mL/L of Fungizone (250 μg/mL), 10% FBS, and 10% CCS. Thereafter, immediately after isolation, the RMAECs were subjected to mRNA and protein expression level determinations. For the fluorescent immunocytochemistry experiments, the RMAECs were plated in PBS for 30 min to allow attachment and then fixed in 3.9% PFA at 4 °C.

### 2.9. In Vivo Permeability Assay

At the end of experiments, the rats were anesthetized with isoflurane and injected with Evans blue dye (EBD, 80 mg/kg, i.v.) for 10 min. EBD binds to the plasma proteins and extravasates to the tissue parenchima at the sites of increased vascular permeability. Then, the rats were euthanized and perfused with saline solution via the left ventricle to wash out the excess EBD. The mesentery, kidney, and liver were removed, washed in cold saline solution, gently dried using a paper towel, weighted, and prepared for EBD extraction as reported elsewhere [29,32]. Briefly, the organs were weighted and homogenized with 100% trichloroacetic acid in a 1:2 (mg:mL) proportion. The homogenates were centrifuged at 4180× *g* for 30 min, and the supernatant optical density was determined spectrophotometrically at 630 nm.

### 2.10. Antioxidant Diet

The antioxidant diet (AoxD) is composed of antioxidant and reductant agents, as follows: 10 g each of freeze-dried oranges, raspberries, grapes, apple, kiwi, carrots, spinach, green tea, pumpkin, and blueberries; 320 mg of mixed tocopherols; 310 mg of vitamin C; 50 μM of GSH; 50 μM of flavonoid genistein; and 50 μM of flavonoid galangin; these were combined with 900 g of PROLAB RMH 3000 (LabDiet, St. Louis, MO, USA) to complete 1 Kg of the AoxD. The fruits and vegetables were purchased at a local supermarket. The mixed tocopherols, vitamin C, GSH, and flavonoid galangin and genistein were purchased from Sigma (USA). The powdered mixture was thoroughly pooled and reconverted into pellets. The AoxD was stored at −20 °C until use. The antioxidant capacity of the AoxD was determined by the Trolox equivalent antioxidant capacity (TEAC) method [33,34]. The reducing ability (reducing power) was determined by the ferric reducing ability of plasma (FRAP) assay [35,36]. The total polyphenol content was also determined [37].

### 2.11. Patients and Volunteers

The study was conducted with 25 patients admitted to the ICU at the Hospital Clínico Metropolitano La Florida, located in Santiago, Chile. The patients were critically ill patients admitted to the ICU with a distributive shock. Furthermore, 22 healthy volunteers were enrolled as a control. Supplemental Table S1 shows the demographic characteristics and clinical data of the patients. This study was approved by the local institutional Ethics and Bioethics Review Board (N° 141008). Furthermore, the Commission of Bioethics and Biosafety of Universidad Andres Bello also approved all the experimental protocols (N°002/2020). The investigation conforms to the principles outlined in the Declaration of Helsinki. All the participants or their surrogates signed an informed consent form prior to entry into the study.

The inclusion criteria for the ICU patients included age > 18 y.o., without restriction for resuscitation and not suffering from shock, defined operationally as a requirement for a norepinephrine (NE) dose > 0.1 g·kg^−1^·min^−1^ to maintain the mean arterial pressure between 65 mmHg and 80 mmHg and a lactate concentration > 4 mmol/L. Additionally, the patients had respiratory support with invasive mechanical ventilation and a C-reactive protein level greater than or equal to 15 mg/dL. These criteria had to be met 48 h after the patients were admitted to the ICU. Clinical decisions were made at the discretion of the attending ICU physicians. The recruitment of patients from both groups was performed consecutively.

The exclusion criteria for the ICU patients included solid cancer with a more advanced stage than carcinoma in situ, lymphoma, leukemia, pregnancy, organ transplantation, red blood cell transfusion with more than 2 units within the previous 48 h after ICU admission, chronic dialysis, liver cirrhosis, nephrotic syndrome, and congestive heart failure. The patients who required drugs that modified coagulation, fibrinolysis, and platelet aggregation were excluded from the study. The patients with chemotherapy, hospitalization, or surgery within the 3 months prior to ICU admission were also excluded. Likewise, the patients on chronic anticoagulant treatment, and the users of aspirin or other antiplatelet drugs in the previous 14 days were excluded from this study.

The healthy volunteers were recruited from the outpatients in the hospital area and research facilities at Universidad Andres Bello. The abovementioned exclusion criteria were also applied to the volunteers. The operational definition of a healthy volunteer was a person without any known chronic disease and explicitly without arterial hypertension, diabetes, body mass index > 30 kg/m^2^, smoking, chronic allergic condition, and pregnancy. In addition, those subjects with an episode of hospitalization or surgery in the previous 3 months prior to enrollment in the study were excluded.

The demographic, clinical, and laboratory data were carefully recorded and collected. The Acute Physiology and Chronic Health Evaluation II (APACHE II) score was evaluated after admission to the ICU. The Sequential Organ Failure Assessment (SOFA) score was determined on the day of blood re-collection. The plasma oxHDL concentration was determined using the Human Oxidized High Density Lipoprotein ELISA Kit (MyBiosource, San Diego, CA, USA), in accordance with the manufacturer’s instructions. The management and the treatment of the patients were carried out by the attending physicians at the ICU without any specific intervention for the purpose of this study. Twenty-eight-day mortality was also recorded.

### 2.12. CECs Separation and Protein Expression Determination by Flow Cytometry

Human circulating endothelial cells (CECs), including circulating endothelial mature cells (CMECs) and circulating endothelial progenitor cells (CEPCs), were separated from the blood samples obtained from the ICU patients 48 to 72 h after admission to the ICU and from the HVs. Blood samples were collected in a 3 mL vacutainer tube containing liquid tripotassium ethylenediaminetetraacetic acid (EDTA) as an anticoagulant. The collection of blood samples and the isolation of cells and their analysis were carried out by two personnel who were blinded to the patient information, as well as to the clinical characteristics or further outcome of the patients. The CMECs and CEPCs were isolated by magnetic bead-based immunoseparation, as described previously [30,38]. Briefly, after the blood samples were obtained, the total mononuclear blood cell fraction was isolated from the blood by Ficoll-Histopaque (Sigma Chemical Co, St Louis, MO, USA) gradient separation. The mononuclear cell fraction was washed by centrifugation with phosphate-buffered saline solution. Then, the mononuclear blood cell fraction was subjected to immunomagnetic bead capture (IBC) using a bead-conjugated CD133 monoclonal antibody and a magnetic cell separation system (Miltenyi Biotec, Bergisch Gladbach, Germany). The captured cells corresponded to an enriched CEPC sample (positive selection, CD133^+^), while the cells contained in the eluted solution contained CMECs (negative selection, CD133^−^). To directly isolate the CMECs, the eluted fluid was subsequently subjected to a second step of IBC positive selection using a bead-conjugated CD146 monoclonal antibody (Miltenyi Biotec), obtaining an enriched CMEC sample (CD146^+^ and CD133^−^). The CMEC and CEPC quantification was performed by flow cytometry. Compensation particles (BD CompBeads) and amine polymer microspheres (Becton Dickinson) were used for compensation [30,38]. Fluorescent-conjugated antibodies against VE-cadherin^+^ and CD31^+^ and against VEGFR-2^+^ and CD34^+^ were used for the detailed phenotype characterization of the CMECs and CEPCs, respectively. Flow cytometry analysis was performed to determine the expression changes of VE-cadherin, α-SMA, and FN, using corresponding monoclonal antibodies (all from R&D Systems, Inc.) coupled to suitable secondary antibodies conjugated to fluorophores (all from ThermoFisher). The labeled cells were then analyzed immediately by flow cytometry (BD FACS Fortessa, BD Biosciences, San José, CA, USA). Color compensation matrices were calculated for each staining combination within each experiment using a single-stained antibody. In all the analyses, doublets and clusters were eliminated. A minimum of 10,000 events were analyzed.

### 2.13. Reagents and Inhibitors

The following reagents and inhibitors were used: LOX-1 inhibitor κ-carrageenan (250 μg/mL, Sigma-Aldrich), neutralizing anti-LOX antibody (1:50, Abcam, Cambridge, UK), NAD(P)H oxidase inhibitor, diphenyleneiodonium (DPI, 10 μM, Sigma-Aldrich), NAD(P)H oxidase inhibitor, apocynin (Apo, 10 mM, Sigma-Aldrich), a reduced form of glutathione (GSH, 1 mM, Sigma-Aldrich), cell permeable antioxidant N-Acetylcysteine (NAC, 5 mM, Tocris, Bristol, UK), NF-κB inhibitor (SC-3060, 5 μM, Santa Cruz Biotechnology, Dallas, TX, USA), JSH-23 (30 μM, Sigma-Aldrich, USA), ALK5 inhibitor, GW-788388 (5 μg/mL) and SB-431542 (30 μM) (MedChemExpress, Monmouth Junction, NJ, USA), Smad3 inhibitor SIS3 (10 μM) and (E)-SIS3 (5 μM) (MedChemExpress, USA), and FITC-dextran 40 kDa (0.5 mg/mL, Sigma-Aldrich, USA). All the inhibitors were added 1 h before and were maintained throughout the treatment. The buffers and salts were purchased from Merck Biosciences.

### 2.14. Statistical Analysis

The results are presented as mean ± SD or mean ± 95% confidence interval (CI) for the relative risk. Differences were considered significant at *p* < 0.05. Statistical differences were assessed by the Student’s *t*-test (or Mann–Whitney test) and one-way analysis of variance (one-way ANOVA) (or Kruskal–Wallis test), followed by Dunn’s post hoc test and two-way analysis of variance (two-way ANOVA), followed by Tukey’s post hoc test. See the figure legends for the specific test used. The relationships between the variables were assessed by means of correlation analysis using Spearman’s correlation coefficients and linear regression. The survival Kaplan–Meier curves were compared by the log-rank (Mantel–Cox) test and the Gehan–Breslow–Wilcoxon test to determine the survival rates. Contingency analyses with Fisher’s exact test were used to assess the relative risk of death. Statistical testing was two-sided and used the 5% significance level. The data were analyzed with GraphPad Prism version 9.4 (GraphPad Software, LLC, San Diego, CA, USA). The samples used in the study were defined to identify the mean magnitude effect of a ≥2-fold change with standard deviations of 10%. Accordingly, a sample size of 16 rats per group, and 25 patients and 22 HVs, would provide 90% statistical power using a two-sided 0.05 significance level.

## 3. Results

### 3.1. OxHDL Induces Endothelial Fibrosis through the LOX-1/NOX-2/ROS Pathway

**Figure 1:**
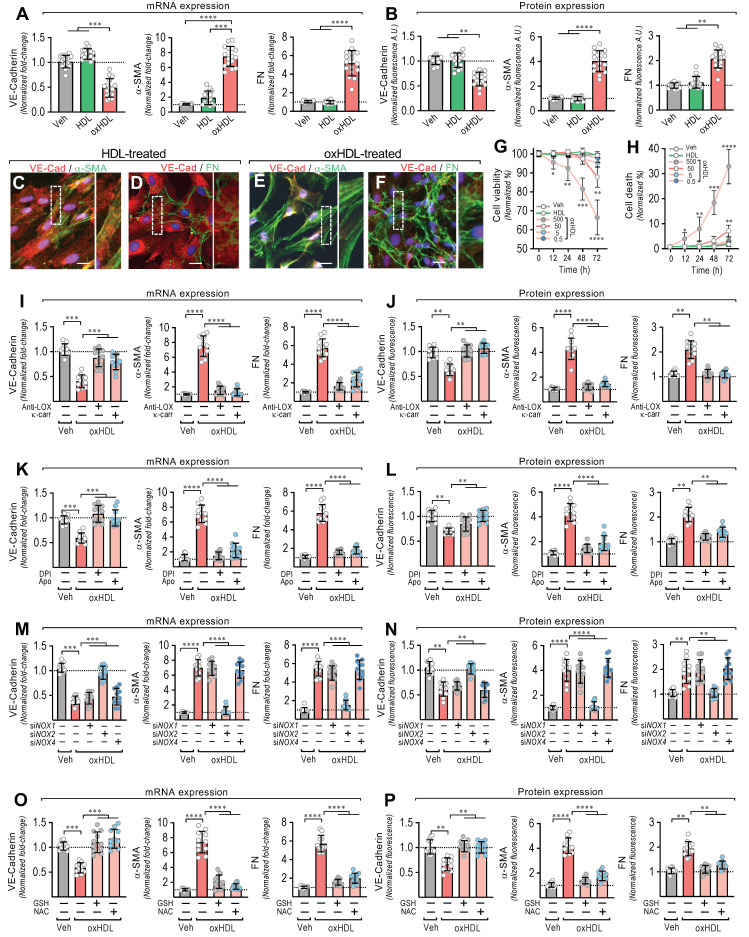
OxHDL induces endothelial fibrosis through the LOX-1/NOX-2/ROS pathway. (**A**,**B**) ECs were exposed to vehicle, HDL 50 μg/mL, and oxHDL 50 μg/mL for 48 h and mRNA (**A**) and the protein (**B**) expression of VE-cadherin (left panels), α-SMA (middle panels), and FN (right panels) was measured. (N = 16). Representative images of cultured ECs treated with HDL 50 μg/mL (**C**,**D**) and oxHDL 50 μg/mL (**E**,**F**) for 48 h, subjected to fluorescent immunocytochemistry experiments performed for the detection of VE-cadherin (red), α-SMA (green), and FN (green). Scale bar represents 50 mm. (N = 5). (**G**,**H**) Viability (**G**) and cell death (**H**) assay in ECs treated with vehicle, HDL (50 μg/mL), and oxHDL (0.5, 5, 50, 500 μg/mL) for 0, 12, 24, 48, and 72 h. (N = 16). (**I**,**J**) ECs were exposed to vehicle and oxHDL 50 μg/mL for 48 h in the absence or presence of the anti-LOX-1 neutralizing antibody (Anti-LOX, 1:50) and the LOX-1 blocker κ-carrageenan (κ-carr, 250 μg/mL), and mRNA (**I**) and protein (**J**) expression of VE-cadherin (left panels), α-SMA (middle panels), and FN (right panels) was measured. (N = 11). (**K**,**L**) ECs were exposed to vehicle and oxHDL 50 μg/mL for 48 h in the absence or presence of the NOX inhibitors DPI (10 μM) and apocynin (Apo, 10 mM), and mRNA (**K**) and protein (**L**) expression of VE-cadherin (left panels), α-SMA (middle panels), and FN (right panels) was measured. (N = 11). (**M**,**N**) ECs were transfected with specific siRNA against NOX-1 (siNOX-1), NOX-2 (siNOX-2), and NOX-4 (siNOX-4) or non-transfected for 72 h, and then, cells were exposed to vehicle and oxHDL 50 μg/mL for 48 h, and mRNA (**M**) and protein (**N**) expression of VE-cadherin (left panels), α-SMA (middle panels), and FN (right panels) was measured. (N = 11). (**O**,**P**) ECs were exposed to vehicle and oxHDL 50 μg/mL for 48 h in the absence or presence of glutathione (GSH, 1 mM) and N-Acetylcysteine (NAC, 5 mM), and mRNA (**O**) and protein (**P**) expression of VE-cadherin (left panels), α-SMA (middle panels), and FN (right panels) was measured. (N = 11). Statistical differences were assessed by one-way analysis of variance (ANOVA) (Kruskal–Wallis), followed by Dunn’s post hoc test, and by two-way ANOVA, followed by Dunnett’s post hoc test for (**G**,**H**). *, *p* < 0.05; **, *p* < 0.01; ***, *p* < 0.001; ****, *p* < 0.0001. Results are expressed as the mean ± SD.

To test whether oxHDL promotes the EndMT process, the ECs were exposed to the vehicle, HDL (50 μg/mL), and oxHDL (50 μg/mL) for 48 h, and the changes in the expression of the endothelial and fibrotic markers were assessed. The oxHDL-treated ECs showed a decreased mRNA and protein expression of VE-cadherin (Figure 1A,B, left panels), whereas the fibrotic marker, α-SMA, was strongly increased at the mRNA and protein levels (Figure 1A,B, middle panels), compared with the vehicle- and HDL-treated cells. One of the main features of fibrosis is the increased production of ECM proteins. Thus, the mRNA expression of the ECM protein fibronectin (FN) was measured. The oxHDL-treated ECs showed an increased mRNA expression of FN (Figure 1A right panel). To investigate the FN expression at the protein level, the secreted fibronectin protein levels in the supernatant were measured. The oxHDL-treated ECs showed an increased FN secretion (Figure 1B, right panel). Moreover, the oxHDL-treated ECs showed an increased mRNA and protein expression of type I and III collagen (Supplemental Figure S1).

Furthermore, immunocytochemistry experiments were performed to study the effect of oxHDL-induced EndMT on cell morphology and cellular protein distribution. The HDL-treated ECs showed a clear expression of VE-cadherin (Figure 1C,D), while the α-SMA (Figure 1C) and FN (Figure 1D) expression was only weakly detected, which is consistent with the endothelial pattern of the basal expression of α-SMA and FN [23]. Additionally, the ECs displayed a cobblestone appearance with a round short-spindle morphology. Similar results were observed in the vehicle-treated cells (not shown). Notably, the oxHDL-treated ECs showed a severe VE-cadherin downregulation (Figure 1E,F). The EC monolayer morphology was disrupted, resulting in a loss of cell-to-cell contacts, which is consistent with the downregulation of the endothelial adhesion protein VE-cadherin. In addition, α-SMA was strongly expressed (Figure 1E), establishing stress fiber configurations, which are representative of the fibrotic phenotype. Furthermore, the expression of FN was enhanced (Figure 1F), as indicated by an extensive extracellular net covering the ECs. In the timeframe of the experiments and the oxHDL dose used, no significant changes in either cell viability (Figure 1G) or cell death were detected (Figure 1H). Endotoxin and inflammatory molecules promote EndMT. Considering that the source of oxHDL used in this work was native HDL, we tested whether the HDL challenge alone was able to elicit an inflammatory response due to contamination with endotoxin or inflammatory molecules. The results showed that the native HDL and the oxidative solution used to oxidize the HDL did not generate any significant difference in the mRNA expression and protein secretion of the pro-inflammatory cytokines TNF-α, IL-1β, and IL-6 (Supplemental Figure S2), indicating that HDL, and consequently the generated oxHDL, have no relevant contamination with inflammatory agents. 

Next, we were prompted to study the underlying molecular mechanism involved in oxHDL-induced endothelial fibrosis. Considering that oxHDL has been reported to bind to LOX-1, we tested whether LOX-1 participates in endothelial fibrosis induced by oxHDL. Thus, the ECs were treated with anti-LOX-1 as a neutralizing antibody and with the LOX-1 blocker κ-carrageenan (κ-carr), and the VE-cadherin, α-SMA, and FN expressions were measured. The incubation of the oxHDL-treated ECs with anti-LOX-1 and κ-carrageenan completely inhibited the oxHDL-induced expression changes in VE-cadherin, α-SMA, and FN at the mRNA (Figure 1K) and protein (Figure 1L) levels, suggesting that LOX-1 is required to elicit oxHDL-induced fibrosis. As LOX-1 stimulates NAD(P)H oxidase (NOX) activation, we evaluated whether the NOX enzyme participates in endothelial fibrosis induced by oxHDL. The incubation of oxHDL-treated ECs with the NOX inhibitors DPI and apocynin completely abolished the oxHDL-induced expression changes in VE-cadherin, α-SMA, and FN at the mRNA (Figure 1K) and protein (Figure 1L) levels. To define the endothelial NOX isoform involved, we performed siRNA-based expression downregulation against the endothelial NOX isoforms: NOX-1, NOX-2, and NOX-4 [39,40]. The oxHDL-treated ECs transfected with siRNA against NOX-2 showed no changes in the expression of VE-cadherin, α-SMA, or FN at the mRNA (Figure 1M) or protein (Figure 1N) levels compared with vehicle-treated cells, while the ECs transfected with siRNA against NOX-1 and NOX-4 showed no inhibition of the oxHDL-induced mRNA or protein expression change. Considering that NOX activation indicates that ROS generation could be involved in oxHDL-induced fibrosis, experiments were performed using the reducing agent GSH and the antioxidant NAC. The oxHDL-treated ECs in the presence of GSH and NAC showed a complete inhibition of the oxHDL-induced expression changes in VE-cadherin, α-SMA, and FN at the mRNA (Figure 1O) and protein (Figure 1P) levels. These results indicate that oxHDL induces endothelial fibrosis through the LOX-1/NOX-2/ROS intracellular pathway.

### 3.2. OxHDL Induces Endothelial Fibrosis through the TGF-β 1/2 Secretion/ALK-5/Smad Protein Pathway

**Figure 2:**
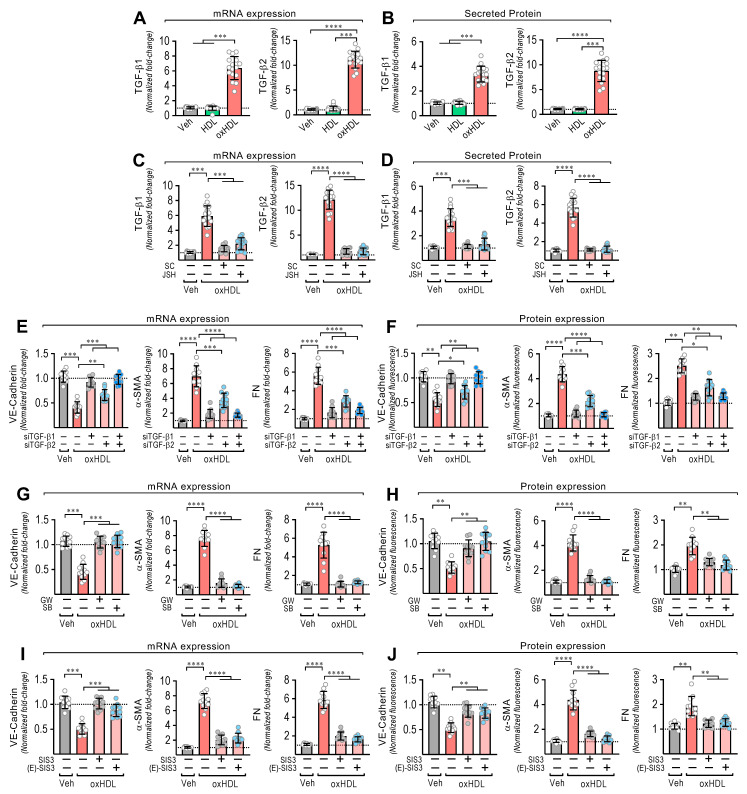
OxHDL induces endothelial fibrosis through the TGF-β1/2 secretion/ALK-5/Smad protein pathway. (**A**,**B**) ECs were exposed to vehicle, HDL 50 μg/mL, and oxHDL 50 μg/mL for 48 h, and mRNA expression (**A**) and protein secretion (**B**) of TGF-β1 (left panels) and TGF-β2 (right panels) were measured. (**C**,**D**) ECs were exposed to vehicle and oxHDL 50 μg/mL for 48 h in the absence or presence of the NF-κB inhibitors SC-3060 (5 μM) and JSH-23 (30 μM), and mRNA expression (**C**) and protein secretion (**D**) of TGF-β1 (left panels) and TGF-β2 (right panels) were measured. (N = 11). (**E**,**F**) ECs were transfected with specific siRNA against TGF-β1 (siTGF-β1) and TGF-β2 (siTGF-β2) or non-transfected for 72 h, and then, cells were exposed to vehicle and oxHDL 50 μg/mL for 48 h, and mRNA (**E**) and protein (**F**) expression of VE-cadherin (left panels), α-SMA (middle panels), and FN (right panels) was measured. (N = 16). (**G**,**H**) ECs were exposed to vehicle and oxHDL 50 μg/mL for 48 h in the absence or presence of the ALK-5 inhibitor GW-788388 (5 μg/mL) and SB-431542 (30 μM), and mRNA (**G**) and protein (**H**) expression of VE-cadherin (left panels), α-SMA (middle panels), and FN (right panels) was measured. (N = 16). (**I**,**J**) ECs were exposed to vehicle and oxHDL 50 μg/mL for 48 h in the absence or presence of the Smad3 inhibitor SIS3 (10 μM) and (E)-SIS3 (5 μM), and mRNA (**I**) and protein (**J**) expression of VE-cadherin (left panels), α-SMA (middle panels), and FN (right panels) was measured. (N = 16). Statistical differences were assessed by one-way analysis of variance (ANOVA) (Kruskal–Wallis) followed by Dunn’s post hoc test. *, *p* < 0.05; **, *p* < 0.01; ***, *p* < 0.001; ****, *p* < 0.0001. Results are expressed as the mean ± SD.

EndMT is induced by TGF-β1 and TGF-β2 [21,22]. Thus, we wondered whether oxHDL stimulates TGF-β1 and TGF-β2 expression and secretion to mediate oxHDL-induced EndMT. The oxHDL-treated ECs showed an increased mRNA expression (Figure 2A) and secreted protein levels (Figure 2B) of TGF-β1 and TGF-β2. Considering that NF-κB promotes TGF-β1/2 expression and that LOX-1 activates NF-κB, we tested whether NF-κB mediates the TGF-β1/2 expression induced by oxHDL. The oxHDL-treated ECs in the presence of the NF-κB inhibitors SC-3060 and JSH-23 abolished the expression of the TGF-β1/2 expression at the mRNA (Figure 2C) and secreted protein (Figure 2D) levels. To corroborate the participation of TGF-β1/2 in generating oxHDL-induced endothelial fibrosis, TGF-β1/2 downregulation was performed using specific siRNAs. The oxHDL-treated ECs transfected with siRNA against TGF-β1 and TGF-β2 showed total and partial significant expression changes, respectively, in VE-cadherin, α-SMA, and FN at the mRNA (Figure 2E) and protein (Figure 2F) levels. TGF-β elicits its actions by binding TGF-β receptor II (TβRII), which binds the ALK5 receptor (TβRI) to trigger TGF-β intracellular signaling. Interestingly, the addition of the ALK-5 inhibitors GW-788388 and SB-431542 to the oxHDL-treated ECs completely inhibited the expression changes of VE-cadherin, α-SMA, and FN at the mRNA (Figure 2G) and protein (Figure 2H) levels. ALK5 activates Smad2 and Smad3 proteins, which bind the Smad4 transcription factor to promote the gene transcription related to EndMT [41,42]. OxHDL-treated ECs in the presence of the Smad3 specific inhibitors SIS3 and (E)-SIS3 inhibited the expression changes of VE-cadherin, α-SMA, and FN at the mRNA (Figure 2I) and protein (Figure 2J) levels. These findings suggest that oxHDL induces endothelial fibrosis through NF-κB-mediated TGF-β expression and secretion, which activates the ALK-5 receptor, promoting Smad protein signaling.

### 3.3. OxHDL Induces Endothelial Hyperpermeability through the LOX-1/NOX-2/ROS Pathway

**Figure 3:**
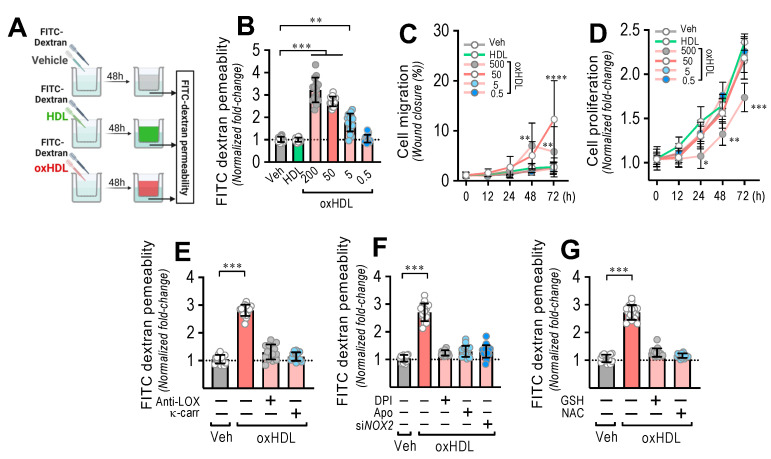
OxHDL induces endothelial hyperpermeability through the LOX-1/NOX-2/ROS pathway. (**A**) Experimental strategy for in vitro oxHDL induces endothelial hyperpermeability determination. Endothelial monolayer was cultured in the upper well in transwell plates and exposed to vehicle, HDL, and oxHDL for 48 h, and FITC-dextran was added to determine permeability in the lower well. (**B**) ECs were exposed to vehicle, HDL (50 μg/mL), and oxHDL (0.5, 5, 50, 500 μg/mL) for 48 h, and endothelial permeability to FITC-dextran was measured. (N = 8). (**C**,**D**) Migration (**C**) and cell proliferation (**D**) assay in ECs treated with vehicle, HDL (50 μg/mL), and oxHDL (0.5, 5, 50, 500 μg/mL) for 0, 12, 24, 48, and 72 h. (N = 16). (**E**) ECs were exposed to vehicle and oxHDL 50 μg/mL for 48 h in the absence or presence of the anti-LOX-1 neutralizing antibody (Anti-LOX, 1:50), and the LOX-1 blocker κ-carrageenan (κ-carr, 250 μg/mL) and endothelial permeability to FITC-dextran was measured. (N = 16). (**F**) ECs were exposed to vehicle and oxHDL 50 μg/mL for 48 h in the absence or presence of the NOX inhibitors DPI (10 μM) and apocynin (Apo, 10 mM), and endothelial permeability to FITC-dextran was measured. ECs were transfected with specific siRNA against NOX-2 (siNOX-2) or non-transfected for 72 h, and then, cells were exposed to vehicle and oxHDL 50 μg/mL for 48 h, and endothelial permeability to FITC-dextran was measured. (N = 16). (**G**) ECs were exposed to vehicle and oxHDL 50 μg/mL for 48 h in the absence or presence of glutathione (GSH, 1 mM) and N-Acetylcysteine (NAC, 5 mM), and endothelial permeability to FITC-dextran was measured. (N = 16). Statistical differences were assessed by one-way analysis of variance (ANOVA) (Kruskal–Wallis) followed by Dunn’s post hoc test, and by two-way ANOVA followed by Dunnett’s post hoc test for C and D. *, *p* < 0.05; **, *p* < 0.01; ***, *p* < 0.001; ****, *p* < 0.0001. Results are expressed as the mean ± SD.

Considering the severe disruption of the endothelium generated as a consequence of oxHDL-induced endothelial fibrosis, to determine whether oxHDL-induced endothelial fibrosis generates hyperpermeability in the endothelial monolayer, we performed transwell assays (Figure 3A). The endothelial monolayer exposed to oxHDL showed a dose-dependent increase in the permeability (Figure 3B), compared to the vehicle- and HDL-treated conditions. Cell migration (Figure 3C) and proliferation (Figure 3D) were not significantly different with different timeframes or oxHDL doses. The oxHDL-treated endothelial monolayer in the presence of anti-LOX-1- and κ-carrageenan did not show increased permeability (Figure 3E), indicating that LOX-1 participates in endothelial hyperpermeability induced by oxHDL. In addition, NOX-2 is also involved in oxHDL-induced endothelial hyperpermeability since the oxHDL-treated ECs in the presence of DPI, Apo, and siRNA against NOX-2 did not show hyperpermeability (Figure 3F). Congruently, the oxHDL-treated ECs in the presence of GSH and NAC abolished the oxHDL-induced increased permeability (Figure 3G). These results indicate that oxHDL induces endothelial hyperpermeability through the LOX-1/NOX-2/ROS pathway.

### 3.4. In Vivo oxHDL Administration Induces Endothelial Fibrosis in Rats

**Figure 4:**
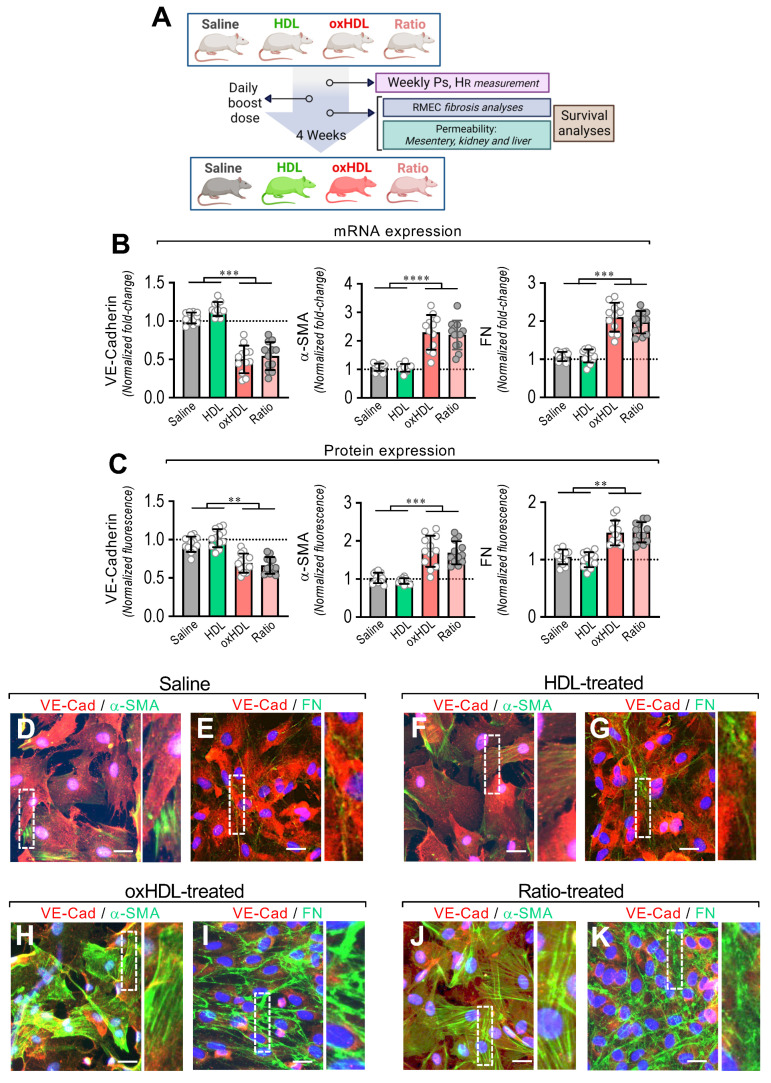
In vivo oxHDL administration induces endothelial fibrosis in rats. (**A**) Experimental strategy for in vivo oxHDL administration in rats and determination of several variables. Rats were treated with saline solution (saline, 0.9% NaCl), HDL (0.4 mg/kg), oxHDL (2 mg/kg), and oxHDL/HDL ratio (0.4/2 mg/kg) by intraperitoneal (I.P.) injection (200 μL) daily for 4 weeks. Systolic pressure (P_S_) and heart rate (H_R_) were determined weekly. At the end of treatment, permeability analysis (mesentery, kidney, and liver) and fibrosis analysis in RMAEC were performed. Survival analyses were performed within the experiments. (**B**,**C**) After saline, HDL, oxHDL, and oxHDL/HDL ratio treatments, RMAECs were extracted and subjected immediately to determination of mRNA (**B**) and protein (**C**) expression of VE-cadherin (left panels), α-SMA (middle panels), and FN (right panels). (N = 12). Statistical differences were assessed by one-way analysis of variance (ANOVA) (Kruskal–Wallis) followed by Dunn’s post hoc test. **, *p* < 0.01; ***, *p* < 0.001; ****, *p* < 0.0001. Results are expressed as the mean ± SD. (**D**–**K**) After saline (**D**,**E**), HDL (**F**,**G**), oxHDL (**H**,**I**), and oxHDL/HDL ratio (**J**,**K**) treatments, RMAECs were extracted and subjected immediately to fluorescent immunocytochemistry for the detection of VE-cadherin (red), α-SMA (green), and FN (green). Scale bar represents 50 mm. (N = 5).

To evaluate whether oxHDL administration generates endothelial fibrosis, the rats were treated with oxHDL (2 mg/kg), HDL (0.4 mg/kg), and saline solution daily for 4 weeks, and the EndMT process and several physiopathological parameters were examined, and survival analysis was performed. A ratio between oxHDL and HDL is present in the ICU patients and is severely increased during systemic inflammatory pathologies because the oxHDL levels rapidly increase, often combined with a decrease in HDL levels. Considering that issue, the rats were also treated with oxHDL and HDL simultaneously, mimicking the oxHDL/HDL ratio (Figure 4A). The HDL and oxHDL doses were able to establish and maintain a constant and reliable lipoprotein plasma level comparable to that observed elsewhere in rats (Supplemental Figure S3).

After treatment, the primary mesenteric endothelial cells (RMAECs) were extracted from the rats of all the groups, and the VE-cadherin, α-SMA, and FN expression was immediately determined. The RMAECs extracted from oxHDL- and the oxHDL/HDL ratio-treated rats showed decreased mRNA and protein expression levels of VE-cadherin (Figure 4B,C, left panels), whereas the fibrotic and ECM markers, α-SMA, and FN were strongly increased at the mRNA and protein levels (Figure 4B,C, middle and right panels), compared with the saline- and HDL-treated rats. Experiments performed in primary rat aortic endothelial cells (RAECs) showed similar results, confiming these findings (Supplemental Figure S4). Moreover, the RMAECs and RAECs extracted from oxHDL- and oxHDL/HDL ratio-treated rats showed increased mRNA and protein levels of type I and III collagen, compared with the saline- and HDL-treated rats (Supplemental Figure S5).

Furthermore, the immunocytochemistry performed in the RMAECs extracted from the saline-treated (Figure 4D,E) and HDL-treated (Figure 4F,G) rats showed a noticeable VE-cadherin (Figure 4D–G) expression, while the α-SMA (Figure 4D,F) and FN (Figure 4E,G) expressions were almost undetectable. In contrast, the RMAECs extracted from the oxHDL-treated (Figure 4H,I) and oxHDL/HDL ratio-treated (Figure 4J,K) rats showed a decreased expression of VE-cadherin (Figure 4H–K), while the α-SMA (Figure 4H,J) and FN (Figure 4I,K) expressions were strongly increased.

### 3.5. In Vivo oxHDL Administration Induces Blood Vessel Hyperpermeability, Hypotension, Increased Risk of Death and Decreased Survival in Rats

**Figure 5:**
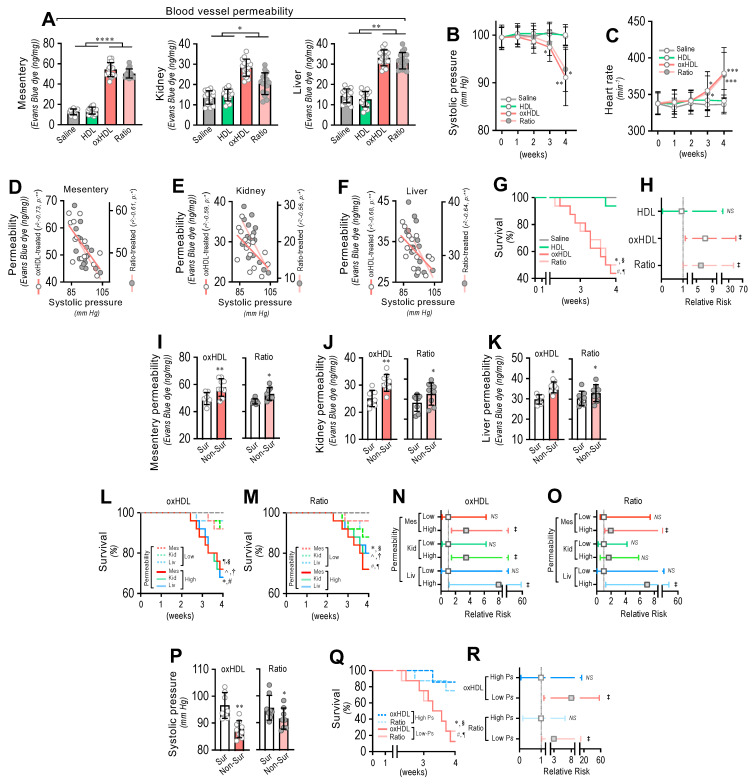
In vivo oxHDL administration induces blood vessel hyperpermeability, hypotension, increased risk of death and decreased survival in rats. (**A**) After saline, HDL, oxHDL, and oxHDL/HDL ratio treatments, mesentery, kidney, and liver were harvested and processed for EBD extraction to determine blood vessel hyperpermeability. EBD content was normalized to total sample weight (N = 16). (**B**,**C**) Systolic pressure (**B**) and heart rate (**C**) recording in saline-, HDL-, oxHDL-, and oxHDL/HDL ratio-treated rats at 1, 2, 3, and 4 weeks. (N = 16). (**D**–**F**) Correlation analyses between systolic pressure with mesentery (**D**), kidney (**E**), and liver (**F**) permeability in oxHDL- and oxHDL/HDL ratio-treated rats. (**G**). Survival (Kaplan–Meier) curves comparing saline-, HDL-, oxHDL-, and oxHDL/HDL ratio-treated rats. * and #, *p* = 0.0003 (log-rank (Mantel–Cox) test) when comparing oxHDL- and oxHDL/HDL ratio-treated rats versus saline-treated (*) and HDL-treated (#) rats. § and ¶, *p* = 0.001 (Gehan–Breslow–Wilcoxon test) when comparing oxHDL- and oxHDL/HDL ratio-treated rats versus saline-treated (¶) and HDL-treated (§) rats. (N = 16). (**H**). Contingency analyses performed to determine relative risk between HDL-, oxHDL-, and oxHDL/HDL ratio-treated rats. ‡, *p* = 0.009 (Fisher’s exact test) compared to saline-treated rats (N = 16). (**I**–**K**). Mesentery (**I**), kidney (**J**), and liver (**K**) permeability in surviving and non-surviving rats from oxHDL- (left panels) and oxHDL/HDL ratio-treated (right panels) groups. (N = 16). (**L**,**M**). Survival (Kaplan–Meier) curves comparing high and low permeability in mesentery, kidney, and liver from oxHDL- (**L**) and oxHDL/HDL ratio-treated (**M**) rats. * and ^, *p* = 0.003 (log-rank (Mantel–Cox) test) when comparing high versus low permeability in kidney and liver, respectively, and #, *p* = 0.009 (log-rank (Mantel–Cox) test) when comparing high versus low permeability in mesentery. § and † *p* = 0.05 (Gehan–Breslow–Wilcoxon test) when comparing high versus low permeability in kidney and liver, respectively, and ¶, *p* = 0.01 (Gehan–Breslow–Wilcoxon test) when comparing high versus low permeability in mesentery. (N = 16). (**N**,**O**). Contingency analyses performed to determine relative risk between high and low permeability in mesentery, kidney, and liver from oxHDL- (**N**) and oxHDL/HDL ratio-treated (**O**) rats. ‡, *p* = 0.008 (Fisher’s exact test) compared to saline-treated rats (N = 16). (**P**) Systolic pressure in surviving and non-surviving rats from oxHDL- (left panels) and oxHDL/HDL ratio-treated (right panels) groups. (N = 16). (**Q**) Survival (Kaplan–Meier) curves comparing high and low systolic pressure from oxHDL- and oxHDL/HDL ratio-treated rats. * and #, *p* = 0.0003 (log-rank (Mantel–Cox) test) when comparing high versus low systolic pressure from oxHDL- and oxHDL/HDL ratio-treated rats. § and ¶, *p* = 0.001 (Gehan–Breslow–Wilcoxon test) when comparing high versus low systolic pressure from oxHDL- and oxHDL/HDL ratio-treated rats. (N = 16). (**R**) Contingency analyses performed to determine relative risk between high and low systolic pressure from oxHDL and oxHDL/HDL ratio-treated rats. ‡, *p* = 0.01 (Fisher’s exact test) compared to saline-treated rats (N = 16). Statistical differences were assessed by one-way analysis of variance (ANOVA) (Kruskal–Wallis), followed by Dunn’s post hoc test and by two-way ANOVA, followed by Dunnett’s post hoc test for B and C. *, *p* < 0.05; **, *p* < 0.01; ***, *p* < 0.001; ****, *p* < 0.0001. Results are expressed as the mean ± SD.

To determine whether oxHDL administration produced blood vessel hyperpermeability, the mesentery, kidney, and liver were extracted from rats and subjected to an Evans blue dye (EBD) permeability assay. After treatment, the mesentery, kidney, and liver extracted from the oxHDL- and oxHDL/HDL ratio-treated rats showed increased EBD accumulation, which indicates blood vessels leaking into the interstitial compartment (Figure 5A), compared with the saline- and HDL-treated rats. The EBD accumulation assays performed in the aorta showed similar results to those shown in Figure 5A, suggesting that edema formation is a broad and systemic process (Supplemental Figure S6).

Often, increased hyperpermeability contributes to a decrease in blood pressure or hypotension. Systolic pressure monitoring showed that the oxHDL- and oxHDL/HDL ratio-treated rats displayed significant hypotension from the third week of treatment (Figure 5B) compared with the saline- and HDL-treated rats. As a compensatory action, the heart rate was found to be increased in the oxHDL- and oxHDL/HDL ratio-treated rats (Figure 5C). Notably, correlation analysis showed that the increased permeability in the mesentery (Figure 5D), kidney (Figure 5E), and liver (Figure 5F) correlated with the change in systolic pressure recorded in the oxHDL- and oxHDL/HDL ratio-treated groups of rats. To evaluate whether the oxHDL treatments affect survival, we analyzed the survival curves within a 4-week time frame. The results showed a significant difference in the oxHDL- and oxHDL/HDL ratio-treated rats compared with the saline- and HDL-treated rats (Figure 5G), as indicated by the log-rank (Mantel–Cox) test. To give more weight to the deaths at early time points, the Gehan–Breslow–Wilcoxon test was performed, which also showed that the oxHDL- and oxHDL/HDL ratio-treated rats had an increased death incidence (Figure 5G). Next, a contingency analysis was performed to determine the relative risk of death. The results showed that the oxHDL and oxHDL/HDL ratio treatment increased the risk of death, whereas the HDL-treated rats showed no difference in the relative risk of death after 4 weeks of treatment (Figure 5H).

Notably, the oxHDL- and oxHDL/HDL ratio-treated non-surviving rats showed higher permeability values in the mesentery (Figure 5I), kidney (Figure 5J), and liver (Figure 5K), compared with the increased permeability values measured in the surviving rats.

Then, we wondered whether increased permeability levels in oxHDL- and oxHDL/HDL ratio-treated rats are associated with death and the risk of death. To assess this hypothesis, we analyzed survival curves within a 4-week timeframe for oxHDL- and oxHDL/HDL ratio-treated rats grouped into high- and low-permeability groups. The high- and low-permeability groups were determined in the oxHDL- and oxHDL/HDL ratio-treated rats using the median permeability value depicted in Figure 5A as the threshold. The survival curve analysis showed significantly decreased survival in the high-permeability groups from the oxHDL-treated (Figure 5L) and oxHDL/HDL ratio-treated (Figure 5M) rats compared with the low-permeability groups, as indicated by the log-rank (Mantel-Cox) test and by the Gehan–Breslow–Wilcoxon test. Contingency analysis showed a significantly increased risk of death in the high-permeability groups from the oxHDL-treated (Figure 5N) and oxHDL/HDL ratio-treated (Figure 5O) rats, whereas the low-permeability groups of rats showed no significant increase in the relative risk of death after 4 weeks of treatment.

Importantly, the oxHDL- and oxHDL/HDL ratio-treated non-surviving rats showed lower systolic pressure values (Figure 5P) than the decreased systolic pressure determinations observed in the surviving rats. This finding is relevant because it denotes that the rats showing lower systolic pressure were more critically ill because of the oxHDL treatment. To delve deeper into this issue, we analyzed the survival curves within a 4-week timeframe for the oxHDL- and oxHDL/HDL ratio-treated rats grouped into high and low systolic pressure values. The high- and low-systolic-pressure groups were determined using the median systolic pressure from Figure 5B as the threshold, based on the value at 4 weeks or the last recording before death. The results showed significantly decreased survival in the low-systolic-pressure group from the oxHDL- and oxHDL/HDL ratio-treated rats compared with the high-systolic-pressure groups, as indicated by the log-rank (Mantel Cox) test and by the Gehan–Breslow–Wilcoxon test (Figure 5Q). Contingency analysis showed a significantly increased risk of death in the low-systolic-pressure groups from the oxHDL- and oxHDL/HDL ratio-treated rats, whereas the high-systolic-pressure groups of rats showed no difference in the relative risk of death after 4 weeks of treatment (Figure 5R).

### 3.6. In Vivo Inhibition of oxHDL-Induced Endothelial Fibrosis Prevented Blood Vessel Hyperpermeability, Hypotension, Death, and Risk of Death in Rats

To generate the in vivo inhibition of oxHDL-induced endothelial fibrosis, the rats were treated with the broadly accepted ALK5 in vivo inhibitor, GW-788388 (5 mg/kg), administered orally by gavage, twice a day, 1 week before and during the oxHDL treatment [43,44,45].

**Figure 6:**
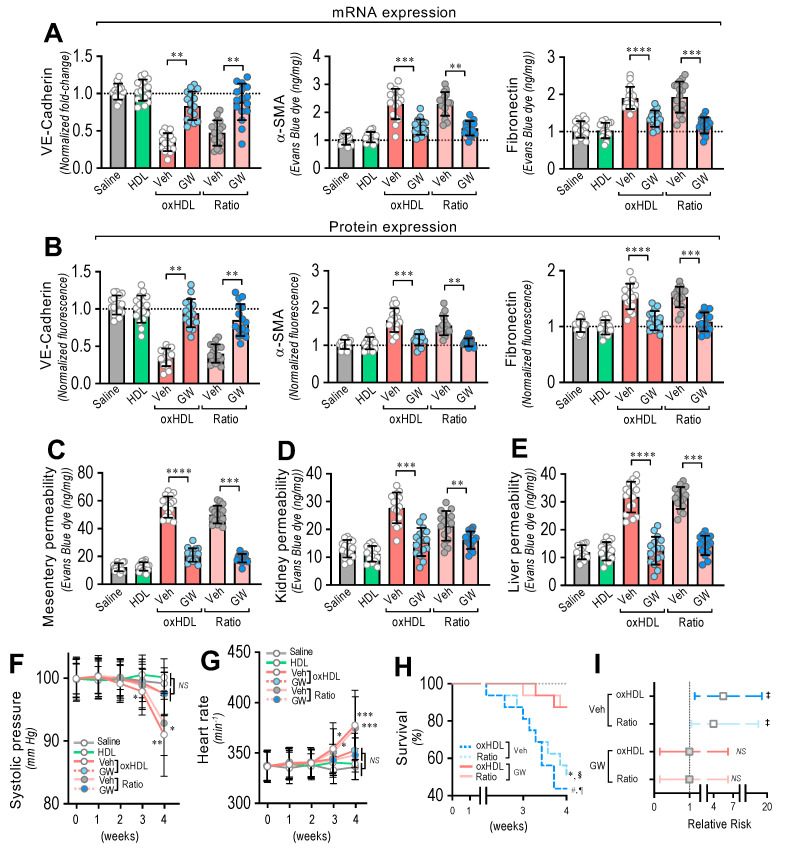
In vivo oxHDL administration induces endothelial fibrosis in rats inhibited by GW-788388. (**A**) After saline, HDL, oxHDL, and oxHDL/HDL ratio treatments, in the presence or absence of GW-788388 administered by gavage (5 mg/kg a day) 1 week before and during the treatment, RMAECs were extracted and subjected immediately to determination of mRNA (**A**) and protein (**B**) expression of VE-cadherin (left panels), α-SMA (middle panels), and FN (right panels). (N = 16). (**C**–**E**) After saline, HDL, oxHDL, and oxHDL/HDL ratio treatments, in the presence or absence of GW-788388 administered by gavage (5 mg/kg a day) 1 week before and during the treatment, mesentery (**C**), kidney (**D**), and liver (**E**) were harvested and processed for EBD extraction to determine blood vessel hyperpermeability. EBD content was normalized to total sample weight (N = 16). (**F**,**G**) Systolic pressure (**F**) and heart rate (**G**) recording in saline, HDL, oxHDL, and oxHDL/HDL ratio treatments, in the presence or absence of GW-788388 administered by gavage (5 mg/kg a day) 1 week before and during the treatment at 1, 2, 3, and 4 weeks. (N = 16). (**H**,**I**). (**H**) Survival (Kaplan–Meier) curves comparing oxHDL and oxHDL/HDL ratio treatments, in the presence or absence of GW-788388 administered by gavage (5 mg/kg a day) 1 week before and during the treatment. * and #, *p* = 0.0003 (log-rank (Mantel–Cox) test) when comparing oxHDL- and oxHDL/HDL ratio-treated rats in the presence of GW versus oxHDL- (*) and oxHDL/HDL ratio-treated (#) rats in the absence of GW. § and ¶, *p* = 0.001 (Gehan–Breslow–Wilcoxon test) when comparing oxHDL- and oxHDL/HDL ratio-treated rats in the presence of GW versus oxHDL- (¶) and oxHDL/HDL ratio-treated (§) rats in the absence of GW. (N = 16). (**I**) Contingency analyses performed to determine relative risk between oxHDL- and oxHDL/HDL ratio-treated rats in the absence or presence GW. ‡, *p* = 0.03 (Fisher’s exact test) compared to saline-treated rats (N = 16). Statistical differences were assessed by one-way analysis of variance (ANOVA) (Kruskal–Wallis) followed by Dunn’s post hoc test and by two-way ANOVA followed by Dunnett’s post hoc test for (**F**,**G**). *, *p* < 0.05; **, *p* < 0.01; ***, *p* < 0.001; ****, *p* < 0.0001. Results are expressed as the mean ± SD.

After treatment, the RMAECs were extracted from the rats, and the VE-cadherin, α-SMA, and FN expression was immediately determined. As we expected, the oxHDL- and oxHDL/HDL ratio-treated rats in the absence of GW-788388 (Veh) showed expression changes in the VE-cadherin, α-SMA, and FN at the mRNA (Figure 6A) and protein (Figure 6B) levels, consistent with oxHDL-induced endothelial fibrosis. Importantly, the RMAECs extracted from the oxHDL- and oxHDL/HDL ratio-treated rats treated with GW-788388 (GW) showed protection against mRNA and protein expression decreases in VE-cadherin (Figure 6A,B, left panels), showing similar expression levels to those observed in the saline- and HDL-treated rats. Similarly, the oxHDL-induced increase in the expression of α-SMA and FN was inhibited at the mRNA and protein levels (Figure 6A,B, middle and right panels) in the oxHDL- and oxHDL/HDL ratio-treated rats and those treated with GW, depicting equivalent expression levels to those of the saline- and HDL-treated rats. The experiments performed in the RAECs showed similar results, confiming the anti-fibrotic action of GW (Supplemental Figure S7). Moreover, the RMAECs and RAECs extracted from the oxHDL- and oxHDL/HDL ratio-treated rats treated with GW showed protection against mRNA and a protein expression increase in type I and III collagen at the mRNA and protein levels (Supplemental Figure S8), showing equivalent expression levels to those of the saline- and HDL-treated rats.

Furthermore, inhibition of the oxHDL-induced endothelial fibrosis prevented hyperpermeability and hypotension. The EBD permeability analysis revealed that the oxHDL- and oxHDL/HDL ratio-treated rats treated with GW showed protection against hyperpermeability in the mesentery (Figure 6C), kidney (Figure 6D), and liver (Figure 6E) compared to the oxHDL- and oxHDL/HDL ratio-treated rats in the absence of GW (Veh). The EBD accumulation experiments performed in the aorta showed similar GW protective actions (Supplemental Figure S9).

Notably, the systolic pressure monitoring showed that the oxHDL/HDL ratio-treated rats and the rats treated with GW displayed no significant variation in systolic pressure (Figure 6F) compared to the vehicle- and HDL-treated rats. Congruently, the heart rate was also normal in the oxHDL/HDL ratio-treated rats treated with GW (Figure 6G).

To study whether the inhibition of oxHDL-induced endothelial fibrosis has a beneficial action on survival, survival curve analysis was performed. The results showed a significant difference between the oxHDL- and oxHDL/HDL ratio-treated rats treated with GW and the rats with the absence of GW treatment (Veh), as indicated by the log-rank (Mantel–Cox) test and the Gehan–Breslow–Wilcoxon test (Figure 6H). Furthermore, the contingency analysis showed a significantly increased risk of death in the oxHDL- and oxHDL/HDL ratio-treated rats with the absence of GW treatment (Veh), whereas the oxHDL- and oxHDL/HDL ratio-treated rats treated with GW showed no difference in the relative risk of death after 4 weeks of treatment (Figure 6I). 

### 3.7. Antioxidant Diet Consumption Inhibits oxHDL-Induced Endothelial Fibrosis and Prevents Blood Vessel Hyperpermeability, Hypotension, Death, and Risk of Death in Rats

**Figure 7:**
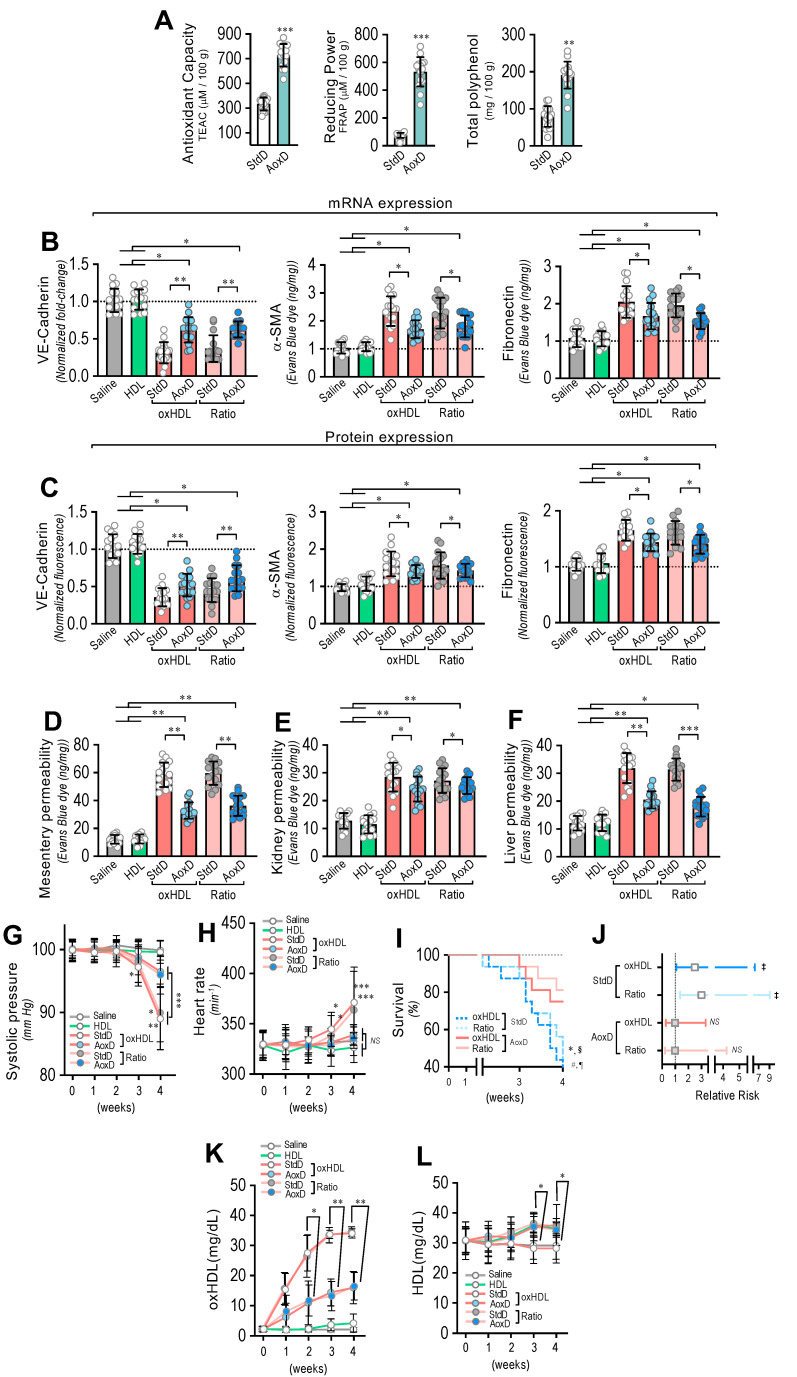
Antioxidant diet consumption inhibits oxHDL-induced endothelial fibrosis and prevents blood vessel hyperpermeability, hypotension, death, and risk of death in rats. (**A**) Antioxidant capacity (left panel), the reducing power (middle panel), and the total polyphenol content (right panel) of antioxidant diet (AoxD) compared to standard diet (StdD). Statistical differences were assessed by Student’s *t*-test (Mann–Whitney). **, *p* < 0.01; ***, *p* < 0.001. Results are expressed as the mean ± SD. (**B**,**C**). After saline, HDL, oxHDL, and oxHDL/HDL ratio treatments fed with the AoxD or StdD for 2 weeks before and during the treatment, RMAECs were extracted and subjected immediately to determination of mRNA (**B**) and protein (**C**) expression of VE-cadherin (left panels), α-SMA (middle panels), and FN (right panels). (N = 15). (**D**–**F**) After saline, HDL, oxHDL, and oxHDL/HDL ratio treatments fed with the AoxD or StdD for 2 weeks before and during the treatment, mesentery (**C**), kidney (**D**), and liver (**E**) were harvested and processed for EBD extraction to determine blood vessel hyperpermeability. EBD content was normalized to total sample weight (N = 16). (**G**,**H**) Systolic pressure (**G**) and heart rate (**H**) recording in saline, HDL, oxHDL, and oxHDL/HDL ratio treatments fed with the AoxD or StdD for 2 weeks before and during the treatment at 1, 2, 3, and 4 weeks. (N = 16). (**I**,**J**). (**I**) Survival (Kaplan–Meier) curves comparing oxHDL and oxHDL/HDL ratio treatments fed with the AoxD or StdD for 2 weeks before and during the treatment. * and #, *p* = 0.0006 (log-rank (Mantel–Cox) test) when comparing oxHDL- and oxHDL/HDL ratio-treated rats fed with AoxD versus oxHDL- (*) and oxHDL/HDL ratio-treated (#) rats fed with StdD. § and ¶, *p* = 0.003 (Gehan–Breslow–Wilcoxon test) when comparing oxHDL- and oxHDL/HDL ratio-treated rats fed with AoxD versus oxHDL- (¶) and oxHDL/HDL ratio-treated (§) rats fed with StdD. (N = 16). (**J**) Contingency analyses performed to determine relative risk between oxHDL- and oxHDL/HDL ratio-treated rats fed with the AoxD or StdD for 2 weeks before and during the treatment. (**K**,**L**). Plasma oxHDL (**K**) and HDL (**L**) determinations in saline, HDL, oxHDL, and oxHDL/HDL ratio treatments fed with the AoxD or StdD for 2 weeks before and during the treatment at 1, 2, 3, and 4 weeks. (N = 16). ‡, *p* = 0.03 (Fisher’s exact test) compared to saline-treated rats (N = 16). Statistical differences were assessed by one-way analysis of variance (ANOVA) (Kruskal–Wallis) followed by Dunn’s post hoc test, and by two-way ANOVA followed by Dunnett’s post hoc test for G, H, K and L. *, *p* < 0.05; **, *p* < 0.01; ***, *p* < 0.001. Results are expressed as the mean ± SD.

Considering that the consumption of both antioxidant and reductant products reduces oxidized molecules, we were prompted to evaluate whether the oral consumption of a diet based on antioxidant and reductant products decreases the deleterious actions induced by oxHDL treatment. To that end, we designed an antioxidant diet (AoxD), and its effects on oxHDL-induced endothelial fibrosis, hyperpermeability, hypotension, mortality, and risk of death were assessed. First, the antioxidant capacity, the reducing power, and the total polyphenol content of the AoxD were determined. The results showed that the AoxD showed increased values of antioxidant capacity, reducing power, and total polyphenol content compared to the standard diet (StdD), and it displayed comparable values to those previously reported for rats fed antioxidant diets (Figure 7A).

The RMAECs were extracted from the rats, and the VE-cadherin, α-SMA, and FN expression was immediately determined. As with the previous results, the oxHDL- and oxHDL/HDL ratio-treated rats fed with StdD showed expression changes in VE-cadherin, α-SMA, and FN at the mRNA (Figure 7B) and protein (Figure 7C) levels, which is consistent with the oxHDL-induced endothelial fibrosis. However, the RMAECs extracted from oxHDL- and oxHDL/HDL ratio-treated rats fed with the AoxD showed significantly reduced changes in the mRNA (Figure 7B) and protein (Figure 7C) expression levels of VE-cadherin, α-SMA, and FN, with expression levels close to those observed in the saline- and HDL-treated rats. The experiments performed in the primary rat aortic endothelial cells (RAECs) showed similar results, confiming the protective action of the AoxD (Supplemental Figure S10). Moreover, the RMAECs and RAECs extracted from oxHDL- and oxHDL/HDL ratio-treated rats fed with the AoxD showed reduced changes in the mRNA and protein expression levels of type I and III collagen (Supplemental Figure S11), showing equivalent expression levels compared with the saline- and HDL-treated rats.

Furthermore, AoxD consumption also prevented oxHDL-induced hyperpermeability and hypotension. The EBD permeability analysis showed that the oxHDL- and oxHDL/HDL ratio-treated rats fed the AoxD showed significant protection against hyperpermeability in the mesentery (Figure 7D), kidney (Figure 7E), and liver (Figure 7F), displaying permeability values similar to those shown in the saline- and HDL-treated rats. The EBD accumulation assays performed in the aorta showed similar AoxD protective actions (Supplemental Figure S12). Importantly, the AoxD consumption showed significant effects on the systolic pressure preservation. The systolic pressure recordings showed that the oxHDL- and oxHDL/HDL ratio-treated rats fed the AoxD showed significant protection against hypotension (Figure 7G), and the heart rate was also found to be normal in these rat groups (Figure 7H) compared to the oxHDL- and oxHDL/HDL ratio-treated rats fed the StdD. Remarkably, the oxHDL-treated rat survival was improved as a consequence of AoxD consumption, probably due to the AoxD-induced preservation of the endothelial phenotype, the permeability, and the blood pressure. The survival curve analysis showed a significant difference between the oxHDL- and oxHDL/HDL ratio-treated rats fed the AoxD and the oxHDL- and the oxHDL/HDL ratio-treated rats fed the StdD, as indicated by the log-rank (Mantel–Cox) test and the Gehan–Breslow–Wilcoxon test (Figure 7I). The contingency analysis showed a significantly increased risk of death in the oxHDL- and oxHDL/HDL ratio-treated rats fed the StdD, whereas the oxHDL- and oxHDL/HDL ratio-treated rats fed the AoxD showed no difference in the relative risk of death after 4 weeks of treatment (Figure 7J).

Next, we wondered whether the AoxD protective actions could be mediated by the capacity to decrease oxHDL plasma levels in oxHDL-treated rats. To test this hypothesis, the HDL and oxHDL plasma levels were measured. Interestingly, the oxHDL- and oxHDL/HDL ratio-treated rats fed the AoxD showed decreased oxHDL levels compared to the rats fed the StdD (Figure 7K). Additionally, the oxHDL- and oxHDL/HDL ratio-treated rats fed the AoxD showed increased HDL levels, reaching values comparable to those of the rats fed the StdD (Figure 7L).

### 3.8. Circulating Endothelial Cells from ICU Patients Show a Fibrotic Expression Pattern, Which Correlates with Plasma oxHDL Levels Associated with Decreased Survival and Increased Risk of Death

**Figure 8:**
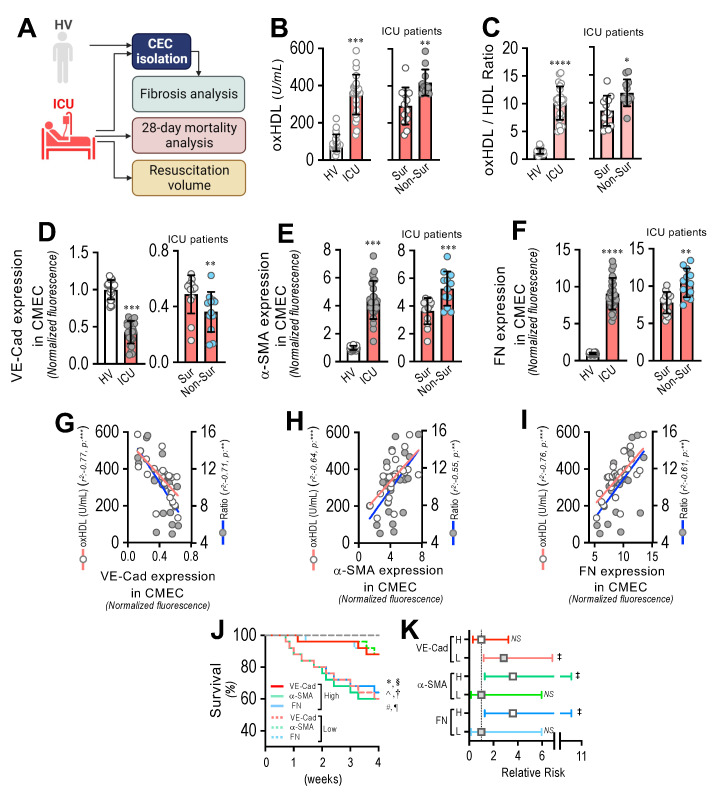
Circulating endothelial cells from ICU patients show a fibrotic expression pattern, which correlates with plasma oxHDL levels associated with decreased survival and increased risk of death. (**A**) Experimental strategy for ICU patients (N = 25) and HVs (N = 22). (**B**,**C**). Plasma oxHDL (**B**) and oxHDL/HDL ratio (**C**) determinations in healthy volunteers (HVs) and ICU patients (left panels) and in surviving and non-surviving ICU patients (right panels). (**D**–**F**). VE-cadherin (**D**), α-SMA (**E**), and FN (**F**) expression in CMECs extracted from healthy volunteers (HVs) and ICU patients (left panels) and in surviving and non-surviving ICU patients (right panels). (**G**–**I**) Correlation analyses between VE-cadherin (**G**), α-SMA (**H**), and FN (**I**) expression in CMECs extracted from ICU patients and oxHDL and oxHDL/HDL ratio determinations. (**J**,**K**). Survival (Kaplan–Meier) curves comparing high and low expression of VE-cadherin, α-SMA, and FN in CMECs from ICU patients *, ^ and #, *p* = 0.006 (log-rank (Mantel–Cox) test) when comparing high versus low expression of VE-cadherin, α-SMA, and FN in CMECs from ICU patients. §, † and ¶, *p* = 0.009 (Gehan–Breslow–Wilcoxon test) when comparing high versus low expression of VE-cadherin, α-SMA, and FN in CMECs from ICU patients. (N = 16) (**J**). Contingency analyses performed to determine relative risk between high and low expression of VE-cadherin, α-SMA, and FN in CMECs from ICU patients. ‡, *p* = 0.008 (Fisher’s exact test) compared to HVs (N = 16) (**K**). Statistical differences were assessed by Student’s *t*-test (Mann–Whitney). *, *p* < 0.05; **, *p* < 0.01; ***, *p* < 0.001; ****, *p* < 0.0001. Results are expressed as the mean ± SD.

To assess the impact of oxHDL-induced endothelial fibrosis in subjects undergoing systemic inflammation, 25 patients admitted to the ICU and 22 healthy volunteers (HVs) were enrolled and several variables were measured (Figure 8A). Supplemental Table S1 shows the demographic characteristics and clinical data of the patients. The patients admitted to the ICU showed increased plasma oxHDL levels compared to the healthy volunteers (HVs) (Figure 8B, left panel). In addition, the oxHDL/HDL ratio was also increased in the ICU patients (Figure 8C, left panel). Interestingly, the plasma oxHDL and the oxHDL/HDL ratio were higher in the non-surviving ICU patients than in the surviving ICU patients (Figure 8B,C, right panels). Importantly, the patients admitted to the ICU exhibited increased circulating endothelial cells (CECs), composed of circulating mature endothelial cells (CMECs), which are a suitable model for studying the vascular ECs of patients and the circulating endothelial progenitor cells (CEPCs) [30,38]. Thus, we were prompted to investigate whether the CMECs from the ICU patients exhibited a protein expression pattern consistent with endothelial fibrosis. The CMECs extracted from the ICU patients showed a decreased protein expression of VE-cadherin (Figure 8D, left panel), whereas the expression levels of the fibrotic and ECM markers α-SMA and FN were strongly increased (Figure 8E,F, left panels) compared to those in the CMECs from the HVs. Furthermore, the VE-cadherin expression was lower in the non-surviving ICU patients than in the surviving ICU patients (Figure 8D, right panel), whereas the α-SMA and FN expression levels were higher in the non-surviving ICU patients than in the surviving ICU patients (Figure 8E,F, right panels).

The correlation analysis showed that the expression of VE-cadherin (Figure 5G), α-SMA (Figure 5H), and FN (Figure 5I) in the CMECs was correlated with the increased oxHDL and oxHDL/HDL ratio levels in the ICU patients.

Next, we analyzed the survival curves within a 4-week timeframe for the ICU patients grouped by the high and low expression of VE-cadherin, α-SMA, and FN. The high- and low-expression VE-cadherin, α-SMA, and FN groups were determined using the median expression value measured in Figure 8D–F as the threshold. The results showed significantly decreased survival curves in the low-expression VE-cadherin group and in the high-expression α-SMA and FN groups, as indicated by the log-rank (Mantel–Cox) test and the Gehan–Breslow–Wilcoxon test (Figure 8J). The contingency analysis showed a significantly increased risk of death in the low-expression VE-cadherin group and in the high-expression α-SMA and FN groups, whereas the high-expression VE-cadherin group and the low-expression α-SMA and FN groups showed no difference in the relative risk of death (Figure 8K).

### 3.9. Resuscitation Fluid Dose Administered to ICU Patients Correlates with Plasma oxHDL and oxHDL/HDL Ratio Levels, Which Is Associated with Decreased Survival and Increased Risk of Death

**Figure 9:**
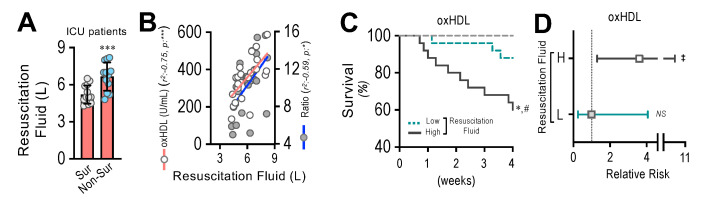
Resuscitation fluid dose administered to ICU patients correlates with plasma oxHDL and oxHDL/HDL ratio levels, which is associated with decreased survival and increased risk of death. (**A**). Resuscitation fluid volume administrated to surviving and non-surviving ICU patients. (**B**) Correlation analyses between resuscitation fluid volume and oxHDL and oxHDL/HDL ratio determinations in ICU patients. (**C**,**D**). Survival (Kaplan–Meier) curves comparing high and low resuscitation fluid volume of ICU patients. *, *p* = 0.001 (log-rank (Mantel–Cox) test) when comparing high versus low resuscitation fluid volume of ICU patients. #, *p* = 0.007 (Gehan–Breslow–Wilcoxon test) when comparing high versus low resuscitation fluid volume of ICU patients. (N = 16) (**C**). Contingency analyses performed to determine relative risk between high and low resuscitation fluid volume of ICU patients. ‡, *p* = 0.007 (Fisher’s exact test) compared to HVs (N = 16) (**D**). Statistical differences were assessed by Student’s *t*-test (Mann–Whitney). *** *p* < 0.001. Results are expressed as the mean ± SD.

By means of endothelial fibrosis, the endothelium loses its integrity, promoting blood vessel hyperpermeability in organs and tissues. Thus, the dose of resuscitation fluid administration to ICU patients denotes the extent of the leaking of blood vessels and the subsequent edema formation. The non-surviving patients admitted to the ICU required the administration of increased doses of resuscitation fluid compared to the surviving patients (Figure 9A). The correlation analysis showed that the administered resuscitation fluid doses correlated with the increased oxHDL and oxHDL/HDL ratio plasma levels from those ICU patients (Figure 9B). The survival curve analyses within a 4-week timeframe for the ICU patients grouped into high and low resuscitation fluid doses were performed. The high- and low-resuscitation-fluid-dose groups were determined using the median value administered to patients as the threshold. The results showed a significant difference in the high-resuscitation-fluid-dose group compared with the low-resuscitation-fluid-dose group, as indicated by the log-rank (Mantel–Cox) test and the Gehan–Breslow–Wilcoxon test (Figure 9C). The contingency analysis showed a significantly increased risk of death in the high-resuscitation-fluid-dose group, whereas the low-resuscitation-fluid-dose group showed no difference in the relative risk of death (Figure 9D).

## 4. Discussion

An enhanced oxidative environment is generated during systemic inflammatory conditions, triggering oxidation in several molecules, including lipids, proteins, and DNA. This oxidative imbalance induces vascular endothelial dysfunction, which is a crucial factor in systemic inflammation pathogenesis. Therefore, elucidating the underlying cellular and molecular mechanisms involved in oxidative stress-mediated vascular endothelial dysfunction and investigating their impact on organ dysfunction and death are crucial to improve the treatment of systemic inflammatory disease. To our knowledge, this is the first report showing that oxHDL is able and sufficient to promote endothelial fibrosis, generating hyperpermeability, hypotension, and increased risk of death. Notably, the inhibition of endothelial fibrosis and antioxidant diet consumption protect against increased oxHDL levels, preserving normal blood pressure, maintaining tissue and organ permeability, and increasing survival.

The participation of HDL as a vascular protective factor has been widely demonstrated [6,46,47]. Notably, Spillmann et al. showed that HDL decreases TGF-β-induced EndMT (47). Moreover, HAECs exposed to HDL did not show a change in the percentage of the ratio of SMA^+^/VE-cadherin^–^ cells and in the collagen III mRNA expression compared to HAECs in the absence of HDL (47). The same results were obtained in our experiments (Supplemental Figures S13 and S14). Interestingly, the HAECs exposed to HDL showed decreased collagen I mRNA expression compared to the HAECs in the absence of HDL (47). Moreover, the same results were obtained in our experiments (Supplemental Figure S14).

In our experiments, an interesting result is that the rats treated with the ratio oxHDL/HDL showed no difference in endothelial fibrosis compared to the rats treated with oxHDL alone, suggesting that HDL decreases its protective role when it is in the presence of oxHDL. This could be due to an inhibitory intracellular cross-talk between the HDL receptor, SR-BI, and the oxHDL receptor LOX-1. It has been reported that the SR-BI activation promotes eNOS-derived NO generation, a well-known oxidative scavenger, and decreases fibrosis in ECs mediated by Akt activation [48,49,50,51]. Interestingly, LOX-1 activation generates the Akt inhibition mediated by the activation of PKCβ, with the consequent reduction in NO production [52,53], contributing to fibrosis. Alternatively, since HDL and oxHDL are very similar molecules, oxHDL could block SR-BI, inhibiting the beneficial HDL signaling by the competition for this receptor. In fact, it has been shown that oxHDL binds SR-BI and the scavenger receptor CD36 [54,55]. Furthermore, oxHDL could promote action in the transcriptional regulation that represses the action of HDL. However, further experiments are needed to elucidate this interesting issue.

However, as a consequence of systemic inflammation, a large amount of ROS is produced, which interacts with native HDL to generate oxHDL, displaying the altered actions involved in the pathophysiological processes [14,56]. Augmented plasma levels of HDL have been related to altered endothelial function [57,58]. In several systemic inflammatory diseases, such as type 2 diabetes mellitus and obesity, increased plasma levels of oxHDL have been observed, while the HDL values are decreased [59,60,61,62], suggesting that excess HDL is transformed into oxHDL, promoting vascular damage. However, these pathological conditions are characterized by the chronic (low and permanent) exposition of circulating oxHDL, which persists for years and decades. Conversely, in this manuscript we focused on the acute actions of oxHDL, characterized by a high and transient level of circulating oxHDL. The ICU patients suffer a sudden burst of oxidative stress caused by an uncontrolled host response against a critical condition. Thus, the acute oxidative burst transforms a massive amount of native HDL into oxHDL, generating a large quantity of circulating oxHDL.

Interestingly, the HDL accessory proteins also accomplish actions in health and disease, including neurological diseases [63,64]. Paraoxonase-1 (PON-1), an accessory protein of HDL, is an enzyme that hydrolyzes oxHDL [65]. An issue of remarkable importance is to investigate whether PON-1 is also able to hydrolyze oxHDL. Further experiments are needed to test that idea. Although the HDL and oxHDL molecules are both present in blood, healthy subjects show almost undetectable oxHDL plasma levels. However, in inflamed patients, the oxHDL plasma level is severely augmented, which increases the plasma oxHDL/HDL ratio. Interestingly, oxHDL and oxHDL/HDL ratio treatment showed similar results, suggesting that oxHDL actions predominate over HDL protection.

OxHDL-induced endothelial fibrosis occurs through LOX-1 signaling, which elicits NOX-2 activation with the resultant generation of ROS. Thus, oxHDL induces positive feedback to enhance the circulating oxidative stress level to reinforce the oxidation of native HDL into oxHDL. This signaling activates NF-κB to promote TGF-β 1/2 secretion, ALK-5 activation, and the subsequent Smad protein activation. The TGF-β/ALK-5/Smad pathway is a reported fibrotic intracellular signaling pathway [22,66,67] that is coupled to oxHDL signaling, providing a robust mechanism for oxHDL-induced endothelial fibrosis. Endothelial fibrosis promotes a change in the protein expression pattern. Interestingly, oxidative stress modulates protein expression in the vasculature [68]. Thus, circulating oxHDL becomes a link between oxidative stress and vascular gene expression.

Systemic inflammatory conditions generate the massive amounts of ROS delivered to tissues and the bloodstream, producing deleterious impacts on vascular function, promoting organ dysfunction, and increasing death [9,69,70]. The ICU patients showed increased oxidative stress levels, which potentially serve as mortality predictors and a severity classification [71,72,73,74], without elucidating the underlying molecular mechanism. Considering these results, circulating oxHDL acts as a molecular mediator between systemic inflammation-induced oxidative stress and hypotension-mediated organ hypoperfusion and death. OxHDL-induced endothelial fibrosis appears to be a molecular and cellular mechanistic pathway based on a change in the endothelial expression profile. Additionally, this finding could explain the ineffectiveness of antibiotic treatments against bacteremia-induced systemic inflammation as they do not limit the generated oxidative stress.

Endothelial dysfunction emerges, at least in part, as a source of organ malfunction during systemic inflammation. However, the endothelial dysfunction mechanism is far from being understood. The endothelium controls several vascular functions, including vascular resistance, hemostasia, and vascular permeability [75]. During systemic inflammation, the endothelial permeability is enhanced, generating vascular leakage and edema in patients [29,30,76,77]. As a result of edema formation, the blood volume into the blood vessels decreases, which provokes the blood pressure to decrease dramatically, increasing the risk of death [30,78,79]. Decreased blood pressure impairs microvascular perfusion, generating massive organ malfunction [76,80]. Therefore, endothelial hyperpermeability is a main factor promoting hypoperfusion-mediated mortality and an increased risk of death [81]. In this context, the downregulation of the endothelial adhesion molecule VE-cadherin strongly contributes to endothelial hyperpermeability by causing the loss of cell-to-cell contacts. This is potentiated by the upregulation of the fibrotic proteins α-SMA and fibronectin, which disrupt the endothelial architecture, contributing to the leakage of the endothelium. Organ and tissue dysfunction is frequently observed in ICU patients and is associated with hypoperfusion induced by decreased blood pressure [82,83,84]. For that reason, hypotensive ICU patients are often treated with the administration of resuscitation fluid, complemented with vasoconstrictors and inotropic agents. However, this strategy has recurrently been insufficient to effectively regulate blood pressure [84,85]. The dose of resuscitation fluid administered to ICU patients is associated with the gravity of the patient’s condition [30,86]. Notably, the oxHDL levels directly correlate with the resuscitation fluid dose, suggesting an association between both parameters. Thus, the results shown herein, indicating that the inhibition of oxHDL-induced endothelial fibrosis by the oral administration of GW-788388 protected against hypotension and decreased death and risk of death, are of outstanding significance. The GW-788388 treatment abolished oxHDL-induced hyperpermeability and significantly increased survival of the oxHDL challenge. GW-788388 has been used to prevent renal and heart fibrosis, and its in vivo application has already been tested [45,87].

Antioxidant agents have been used to prevent and treat systemic inflammatory conditions [88,89,90,91]. However, the underlying mechanism involved in the therapeutic role of antioxidants in ICU patient treatment is poorly understood. Here, AoxD consumption was able to inhibit oxHDL-induced endothelial fibrosis and hyperpermeability. Notably, AoxD consumption prevented hypotension, increased survival, and decreased the risk of death. Notably, AoxD consumption significantly reduced the increased plasma oxHDL levels in oxHDL- and oxHDL/HDL ratio-treated rats. Furthermore, the reduction in plasma HDL levels was fully restored. These findings suggest that the AoxD promotes a reductant-based conversion of oxHDL to HDL, possibly recruiting further antioxidant agents and activating reducing enzymes. However, further experiments are needed to confirm this hypothesis. The AoxD showed a significant increase in antioxidant capacity, reducing power, and total polyphenol content compared to the StdD, which is in accordance with further antioxidant formulations containing vitamin C, tocopherols, and flavonoids [92,93,94]. Remarkably, the beneficial potential of dietary compounds for improving lipid-associated cardiovascular risk has been highlighted in food supplements derived from red seaweed marine algae, where porphyrin and carrageenan, the latter being a LOX-1 antagonist, improved lipid metabolism, reduced oxidative stress, protected against hyperlipidemia, and lowered total and low-density lipoprotein cholesterol [95]. Therefore, determining the minimal formulation to obtain the maximal protective effects is an issue of high interest.

ICU patients, mainly those with sepsis syndrome, exhibit a significant mortality rate, which is associated with organ failure [96,97]. Interestingly, surviving ICU patients exhibit relevant organ malfunction or pathological conditions that are not directly related to the ICU stay [98,99,100]. Taking into consideration that the results shown here demonstrate that oxHDL levels in ICU patients correlate with both CMEC fibrosis and the administered dose of resuscitation fluid, it is reasonable to hypothesize that extensive oxHDL-induced endothelial fibrosis at the organ tissue level contributes to initiating and maintaining organ hypoperfusion in ICU patients and survivors. Undoubtedly, several well-designed experiments are needed to demonstrate these hypotheses.

## 5. Conclusions

Taken together, our results demonstrated that oxHDL generates endothelial fibrosis, which potentially impacts systemic inflammatory disease outcomes. Notably, the inhibition of endothelial fibrosis, as well as antioxidant diet consumption, emerge as therapeutic alternatives against the deleterious effects of increased levels of oxHDL.

## Data Availability

The data presented in this study are available on Supplementary Material or on request from the corresponding authors.

## Supplementary Materials

The following supporting information can be downloaded at: https://www.mdpi.com/article/10.3390/antiox11122469/s1, Supplemental Table S1: demographic characteristics and clinical data of ICU and healthy volunteers, Supplemental Figure S1: oxHDL induces expression of collagen I and III, Supplemental Figure S2: HDL and oxidative solution do not induce pro-inflammatory cytokines TNF-α, IL-1β, and IL-6 expression, Supplemental Figure S3: plasma HDL and oxHDL determinations in rats treated with HDL and oxHDL, Supplemental Figure S4: in vivo oxHDL administration induces endothelial fibrosis in RAECs extracted from rats, Supplemental Figure S5: in vivo oxHDL administration induces endothelial expression of collagen I and collagen III in rats, Supplemental Figure S6: in vivo oxHDL administration induces aorta hyperpermeability in rats, Supplemental Figure S7: in vivo oxHDL administration induces endothelial fibrosis in RAECs extracted from rats, which is inhibited by GW-788388, Supplemental Figure S8: in vivo oxHDL administration induces endothelial expression of collagen I and collagen III in rats, which is inhibited by GW-788388, Supplemental Figure S9: in vivo oxHDL administration induces aorta hyperpermeability in rats, which is inhibited by GW-788388, Supplemental Figure S10: in vivo oxHDL administration induces endothelial fibrosis in RAECs extracted from rats, which is decreased by the antioxidant diet consumption, Supplemental Figure S11: in vivo oxHDL administration induces endothelial expression of collagen I and collagen III in rats, which is inhibited by the antioxidant diet consumption, Supplemental Figure S12: in vivo oxHDL administration induces aorta hyperpermeability in rats, which is inhibited by the antioxidant diet consumption, Supplemental Figure S13: HDL effect on the detection of the percentage of α-SMA-positive and VE-cadherin-negative cells, Supplemental Figure S14: HDL induces decreased collagen I expression.
