# Supplementary material for: Oxidized High-Density Lipoprotein Induces Endothelial Fibrosis Promoting Hyperpermeability, Hypotension, and Increased Mortality
Source: Antioxidants (Basel). 2022 Dec 15;11(12):2469. doi: 10.3390/antiox11122469 (PMC9774523; doi:10.3390/antiox11122469)

Supplemental information

SUPPLEMENTAL MATERIALS AND METHODS

Human artery endothelial cell culture. Detection of mRNA and protein expression of collagen I and II, and pro-inflammatory cytokines TNF-α, IL-1β and IL-6.

Human aortic endothelial cells (HAEC, Lonza, Chicago, USA) were cultured at 37°C in a 5%:95% CO_2:air atmosphere in in EGM-2 medium supplemented with 2% FBS, 2 mM glutamine and 50 U/ml penicillin-streptomycin (Sigma-Aldrich, St Louis, MO, USA). On the day before the study, the FBS concentration was reduced to 1%. RT-qPCR experiments were performed to measure type I and III collagen, TNF-α, IL-1β and IL-6 mRNA levels in ECs. Total RNA was extracted with Trizol according to the manufacturer's protocol (Invitrogen, USA). DNAse I-treated RNA was used for reverse transcription using the Super Script II Kit (Invitrogen, USA). Equal amounts of RNA were used as templates in each reaction. Quantitative-PCR was performed using the SYBR Green PCR Master Mix (AB Applied Biosystems, USA). Assays were run using a RotorGene instrument (Corbet Research, Australia). Data are presented as relative mRNA levels of the gene of interest normalized to relative levels of 28S mRNA and normalized against control condition. Flow cytometry analysis was performed to determine expression changes of collagen I and II, using corresponding monoclonal antibodies (all from R&D Systems, Inc). The labeled cells were then analyzed immediately by flow cytometry (BD FACS Fortessa, BD Biosciences, USA). Color compensation matrices were calculated for each staining combination within each experiment using single-stained antibody. In all analyses, doublets and clusters were eliminated. A minimum of 10,000 events were analyzed. Determination of TNF-α, IL-1β and IL-6 was performed I supernatant by mean of ELISA assay (Adcam, Inc) in according to instructions by the manufacturer. Lipopolysaccharide was purchased in Sigma-Aldrich.

Primary rat aortic endothelial cell (RAEC) isolation.

Primary rat aortic endothelial cells (RAECs) were isolated from the aorta. Aorta on its apical end and cannulated from its distal end with a polyethylene tubing connected to a 21-gauge syringe. Then, aorta was surgically removed and washed with sterile PBS. For the enzymatic isolation of RAECs, aorta was slowly perfused in a culture hood for 5 min with 5 mL M-199 medium supplemented with 40 μL Pen/Strep (10,000 U/mL/10,000 μg/mL), 20 μL Fungizone (250 μg/mL), and 12.5 mg collagenase type II. The cell suspension was centrifuged at 3000 rpm for 7 min; the pellet was reconstituted in 3 mL M-199 medium supplemented with 8 mL/L of Pen/Strep (10,000 U/mL/10,000 μg/mL), 4 mL/L of Fungizone (250 μg/mL), 10% FBS, and 10% CCS. Thereafter, RAECs were subjected immediately before isolation to mRNA and protein expression levels determinations. For fluorescent immunocytochemistry experiments, RAECs were plated in PBS by 30 min to allow attachment and then fixed in 3.9% PFA at 4°C.

In vivo Permeability Assay in the aorta.

At the end of experiments, rats were anesthetized with isoflurane and injected with Evans blue dye (EBD, 80 mg/kg, i.v.) for 10 min. EBD binds to plasma proteins and extravasates to tissue parenchima at sites of increased vascular permeability. Then, rats were euthanized and perfused with saline

solution via the left ventricle to wash excess EBD. The aorta was removed, washed in cold saline solution, gently dried using a paper towel, weighted, and prepared for EBD extraction. Briefly, organs were weighted and homogenized with 100% trichloroacetic acid in a 1:2 (mg:mL) proportion. Homogenates were centrifuged at 4180·g for 30 min and supernatant optical density was determined spectrophotometrically at 630 nm.

HDL and oxHDL measurements in rat.

For determination of plasma HDL and oxHDL concentration, a small sample of blood was obtained by tail puncture weekly from two weeks before treatment and during the 4-weeks treatment and subjected immediately to HDL and oxHDL determination. Plasma HDL and oxHDL concentration was determined using the Rat Oxidized High Density Lipoprotein ELISA Kit (MyBiosource, USA) and the Rat High Density Lipoprotein ELISA Kit (MyBiosource, USA), respectively, in according to instructions by the manufacturer.

SUPPLEMENTAL TABLE LEGENDS

Supplemental Table S1. Demographic characteristics and clinical data of ICU and healthy volunteers

SUPPLEMENTAL FIGURE LEGENDS

Supplemental Figure S1. OxHDL induces expression of collagen I and III. (A-B) ECs were exposed to vehicle, HDL 50 µg/mL and oxHDL 50 µg/mL for 48 h and mRNA (A) and protein (B) expression of collagen I (left panels) and collagen III (right panels) was measured. (N = 16). Statistical differences were assessed by one-way analysis of variance (ANOVA) (Kruskal–Wallis) followed by Dunn's post hoc test. *, $p<0.05$; **, $p<0.01$; ***, $p<0.001$; ****, $p<0.0001$. Results are expressed as the mean ± SD.

Supplemental Figure S2. HDL and oxidative solution do not induce pro-inflammatory cytokines TNF-α, IL-1β and IL-6 expression. ECs were exposed to: vehicle or HDL 50 µg/mL (left panels), vehicle or oxidative solution (middle panels), or vehicle or LPS 1 µg/mL (right panels) for 48 h, and mRNA and protein expression of **TNF-α** (A)**, IL-1β** (B) **and IL-6** (C) was measured. (N = 16). Statistical differences were assessed by one-way analysis of variance (ANOVA) (Kruskal–Wallis) followed by Dunn's post hoc test. *, $p<0.05$; **, $p<0.01$; ***, $p<0.001$; ****, $p<0.0001$. Results are expressed as the mean ± SD.

Supplemental Figure S3. Plasma HDL and oxHDL determinations in rats treated with HDL and oxHDL. Rats were intraperitoneally treated with HDL (0.4 mg/kg), and oxHDL (2 mg/kg) by intraperitoneal (I.P.) injection (200 µL) every 24 h for 4 weeks. After treatment, blood samples (arrows) were collected to determine HDL (**A**) and oxHDL (**B**). (N = 16). Statistical differences were assessed by one-way analysis of variance (ANOVA) (Kruskal–Wallis) followed by Dunn's *post hoc* test. *, $p<0.05$; **, $p<0.01$; ***, $p<0.001$; ****, $p<0.0001$. Results are expressed as the mean ± SD.

Supplemental Figure S4. In vivo oxHDL administration induces endothelial fibrosis in RAECs extracted from rats. After saline, HDL, oxHDL and oxHDL/HDL ratio treatments, RAECs were extracted and subjected immediately to determination of mRNA (B) and protein (C) expression of VE-Cadherin (left panels), α-SMA (middle panels), and FN (right panels). (N = 12). Statistical differences were assessed by one-way analysis of variance (ANOVA) (Kruskal–Wallis) followed by Dunn's post hoc test. **, $p<0.01$; ***, $p<0.001$; ****, $p<0.0001$. Results are expressed as the mean ± SD.

Supplemental Figure S5. In vivo oxHDL administration induces endothelial expression of collagen I and collagen III in rats. After saline, HDL, oxHDL and oxHDL/HDL ratio treatments, RMAECs (A-B) and RAECs (C-D) were extracted and subjected immediately to determination of mRNA (A and C) and protein (B and D) expression of collagen I (left panels) and collagen III (right panels). Statistical differences were assessed by one-way analysis of variance (ANOVA) (Kruskal–Wallis) followed by Dunn's post hoc test. **, $p<0.01$; ***, $p<0.001$. Results are expressed as the mean ± SD.

Supplemental Figure S6. In vivo oxHDL administration induces aorta hyperpermeability in rats. After saline, HDL, oxHDL and oxHDL/HDL ratio treatments, the aorta was extracted and processed for EBD extraction to determine aorta hyperpermeability. EBD content was normalized to total sample weight (N = 16). Statistical differences were assessed by one-way analysis of variance (ANOVA) (Kruskal–Wallis) followed by Dunn's post hoc test. **, $p<0.01$. Results are expressed as the mean ± SD.

Supplemental Figure S7. In vivo oxHDL administration induces endothelial fibrosis in RAECs extracted from rats, which is inhibited by GW-788388. After saline, HDL, oxHDL and oxHDL/HDL ratio treatments, in the presence or absence of GW-788388 administered by gavage (5 mg/kg a day) 1 week before and during the treatment, RAECs were extracted and subjected immediately to determination of mRNA (B) and protein (C) expression of VE-Cadherin (left panels), α-SMA (middle panels), and FN (right panels). (N = 12). Statistical differences were assessed by one-way analysis of variance (ANOVA) (Kruskal–Wallis) followed by Dunn's post hoc test. *, $p<0.05$; **, $p<0.01$; ***, $p<0.001$. Results are expressed as the mean ± SD.

Supplemental Figure S8. In vivo oxHDL administration induces endothelial expression of collagen I and collagen III in rats, which is inhibited by GW-788388. After saline, HDL, oxHDL and oxHDL/HDL ratio treatments, in the presence or absence of GW-788388 administered by gavage (5 mg/kg a day) 1 week before and during the treatment, RMAECs (A-B) and RAECs (C-D) were extracted and subjected immediately to determination of mRNA (A and C) and protein (B and D) expression of collagen I (left panels) and collagen III (right panels). Statistical differences were assessed by one-way analysis of variance (ANOVA) (Kruskal–Wallis) followed by Dunn's post hoc test. *, $p<0.05$; **, $p<0.01$; ***, $p<0.001$. Results are expressed as the mean ± SD.

Supplemental Figure S9. In vivo oxHDL administration induces aorta hyperpermeability in rats, which is inhibited by GW-788388. After saline, HDL, oxHDL and oxHDL/HDL ratio treatments, in the presence or absence of GW-788388 administered by gavage (5 mg/kg a day) 1 week before and during the treatment, the aorta was extracted and processed for EBD extraction to determine aorta hyperpermeability. EBD content was normalized to total sample weight (N = 16). Statistical differences were assessed by one-way analysis of variance (ANOVA) (Kruskal–Wallis) followed by Dunn's post hoc test. *, $p<0.05$; **, $p<0.01$; ***, $p<0.001$. Results are expressed as the mean ± SD.

Supplemental Figure S10. In vivo oxHDL administration induces endothelial fibrosis in RAECs extracted from rats, which is decreased by the antioxidant diet consumption. After saline, HDL, oxHDL and

oxHDL/HDL ratio treatments fed with the AoxD or StdD for 2 weeks before and during the treatment, RAECs were extracted and subjected immediately to determination of mRNA (A) and protein (B) expression of VE-Cadherin (left panels), α-SMA (middle panels), and FN (right panels). (N = 12). Statistical differences were assessed by one-way analysis of variance (ANOVA) (Kruskal–Wallis) followed by Dunn's post hoc test. *, $p<0.05$; **, $p<0.01$. Results are expressed as the mean ± SD.

Supplemental Figure S11. In vivo oxHDL administration induces endothelial expression of collagen I and collagen III in rats, which is inhibited by the antioxidant diet consumption. After saline, HDL, oxHDL and oxHDL/HDL ratio treatments fed with the AoxD or StdD for 2 weeks before and during the treatment, RMAECs (A-B) and RAECs (C-D) were extracted and subjected immediately to determination of mRNA (A and C) and protein (B and D) expression of collagen I (left panels) and collagen III (right panels). Statistical differences were assessed by one-way analysis of variance (ANOVA) (Kruskal–Wallis) followed by Dunn's post hoc test. *, $p<0.05$; **, $p<0.01$; ***, $p<0.001$. Results are expressed as the mean ± SD.

Supplemental Figure S12. In vivo oxHDL administration induces aorta hyperpermeability in rats, which is inhibited by the antioxidant diet consumption. After saline, HDL, oxHDL and oxHDL/HDL ratio treatments fed with the AoxD or StdD for 2 weeks before and during the treatment, the aorta was extracted and processed for EBD extraction to determine aorta hyperpermeability. EBD content was normalized to total sample weight (N = 16). Statistical differences were assessed by one-way analysis of variance (ANOVA) (Kruskal–Wallis) followed by Dunn's post hoc test. *, $p<0.05$; **, $p<0.01$. Results are expressed as the mean ± SD.

Supplemental Figure S13. HDL effect on the detection of the percentage of α-SMA–positive and VE-cadherin–negative cells. HAECs were exposed to vehicle or HDL 50 μg/mL for 48 h and percentage (%) of α-SMA–positive and VE-cadherin–negative was determined. Statistical differences were assessed by student's t-test (Mann-Whitney). Results are expressed as the mean ± SD.

Supplemental Figure S14. HDL induces decreased collagen I expression. ECs were exposed to vehicle or HDL 50 μg/mL for 48 h and mRNA (A) and protein (B) expression of collagen I (left panels) and collagen III (right panels) was measured. (N = 16). Statistical differences were assessed by student's t-test (Mann-Whitney). Results are expressed as the mean ± SD. *, $p<0.05$; **, $p<0.01$. Results are expressed as the mean ± SD.

Supplemental Table S1

Supplemental Table 1. Demographic characteristics and clinical data of ICU patients and healthy volunteers

Variable	ICU Patients (n=25)	Healthy Volunteers (n=22)
Age (ys), median (IQR)	64 (53-76)	57 (44-71)
BMI, median (IQR)	25.3 (23.8-29.7)	23.2 (21.9-28.3)
Male sex, (% of male)	48	53
Time of sampling (h)*, median (IQR)	51 (43 - 62)	NA
APACHE II, median (IQR)	24 (20.8-27)	ND
SOFA, median (IQR)	10.8 (8 - 12.3)	ND
CRP (mg/dl), median (IQR)	28.3 (21.7 - 31.6)	ND
Resuscitation Fluid (Liter),median (IQR)	6.1 (5.2 - 8.8)	NA
Blood lactate level (mmol/L)median (IQR)	7.9 (7.1 - 13.9)	ND
Norepinephrine (ug/kg/min) median (IQR)	0.33 (0.2 - 0.65)	NA
Renal replacement therapy*(%)	32.8	0
Corticosteroids, %	82	NA

Definition of abbreviations: BMI, body mass index; CRP, C-reactive protein; APACHE-II, Acute Physiology and Chronic Health Evaluation II score; SOFA, Sepsis-related Organ Failure Assessment, IQR: interquartile range (expressed as percentile 25th, percentile 75th), ND: non-determined, NA_ non-applicable

* The sample for analysis was collected always prior to connection to a renal replacement therapy.

Supplemental Figure S1

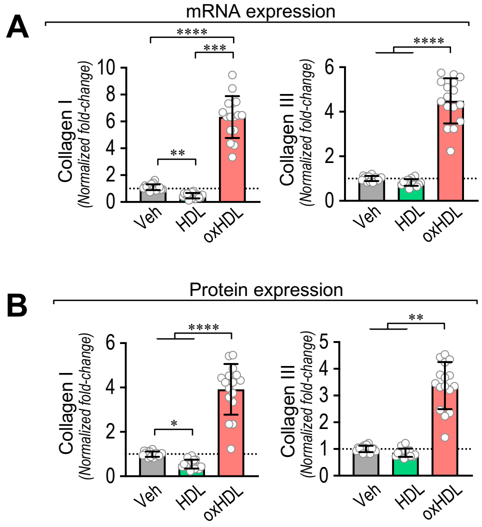

Supplemental Figure S2

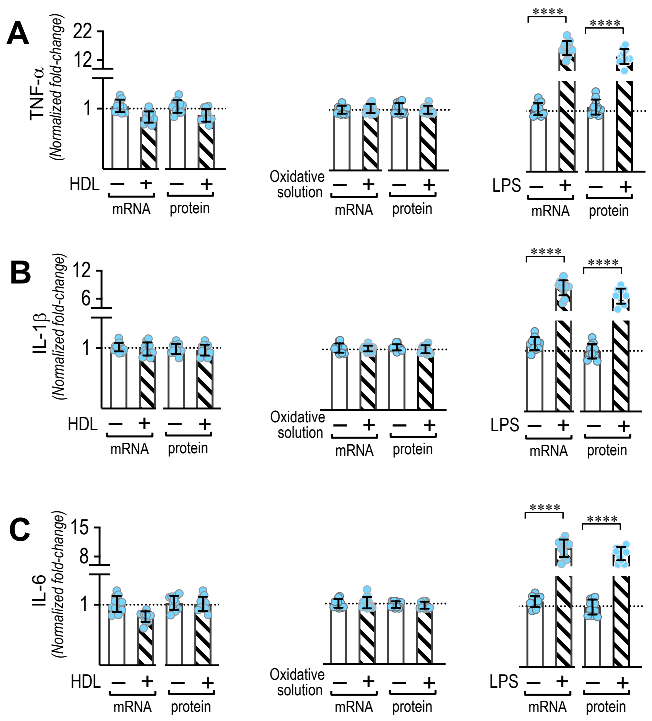

Supplemental Figure S3

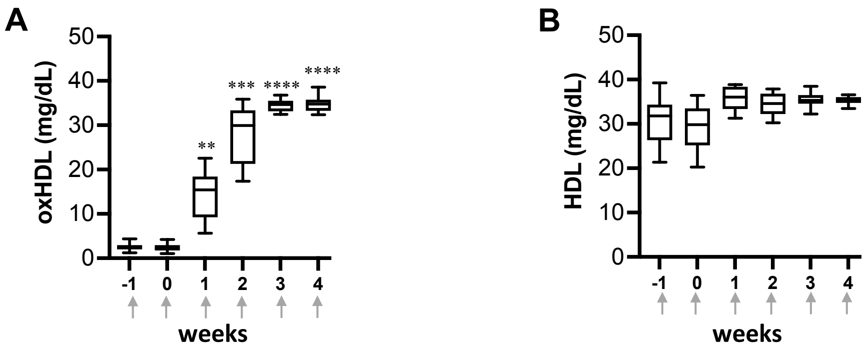

Supplemental Figure S4

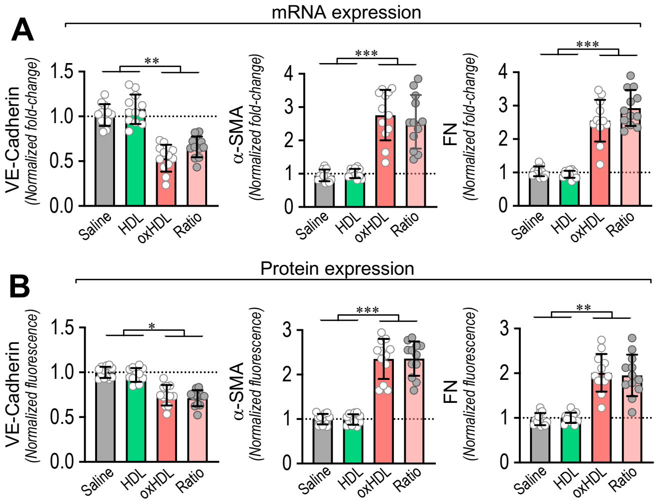

Supplemental Figure S5

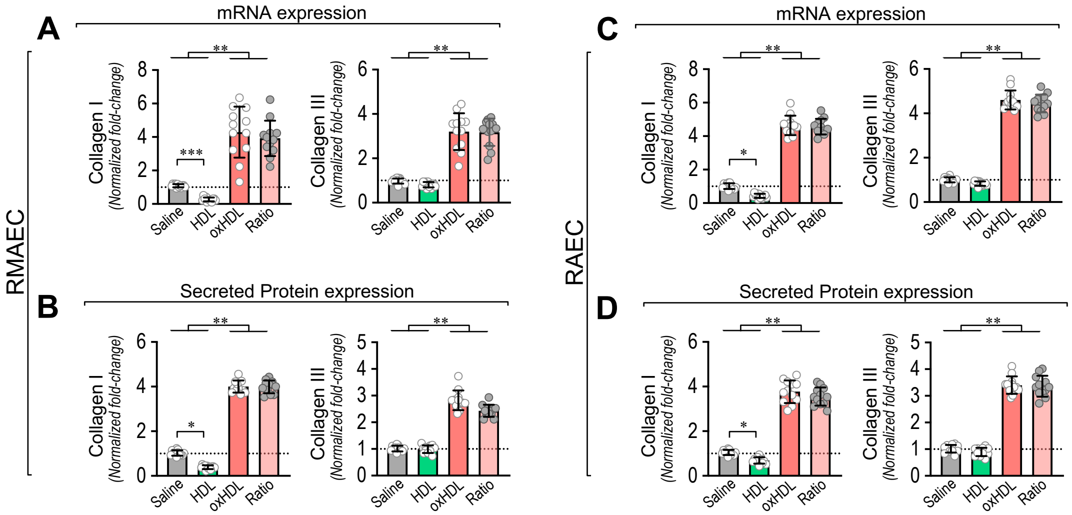

Supplemental Figure S6

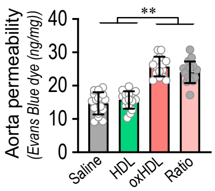

Supplemental Figure S7

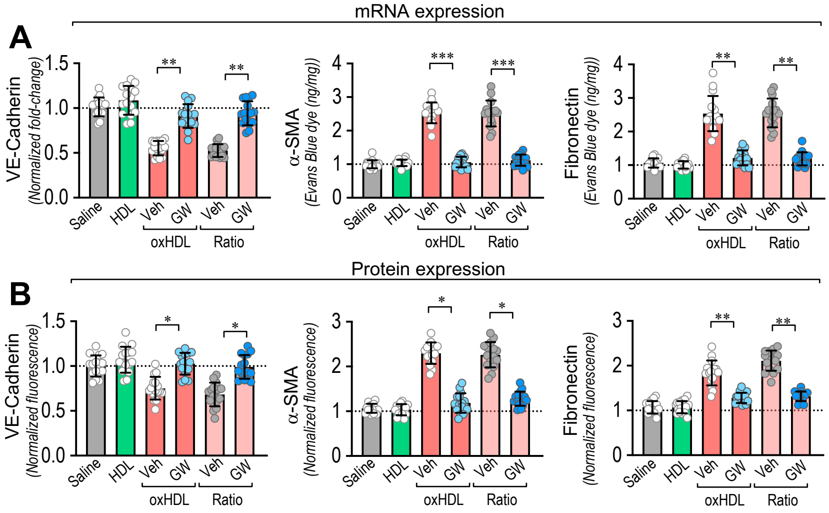

Supplemental Figure S8

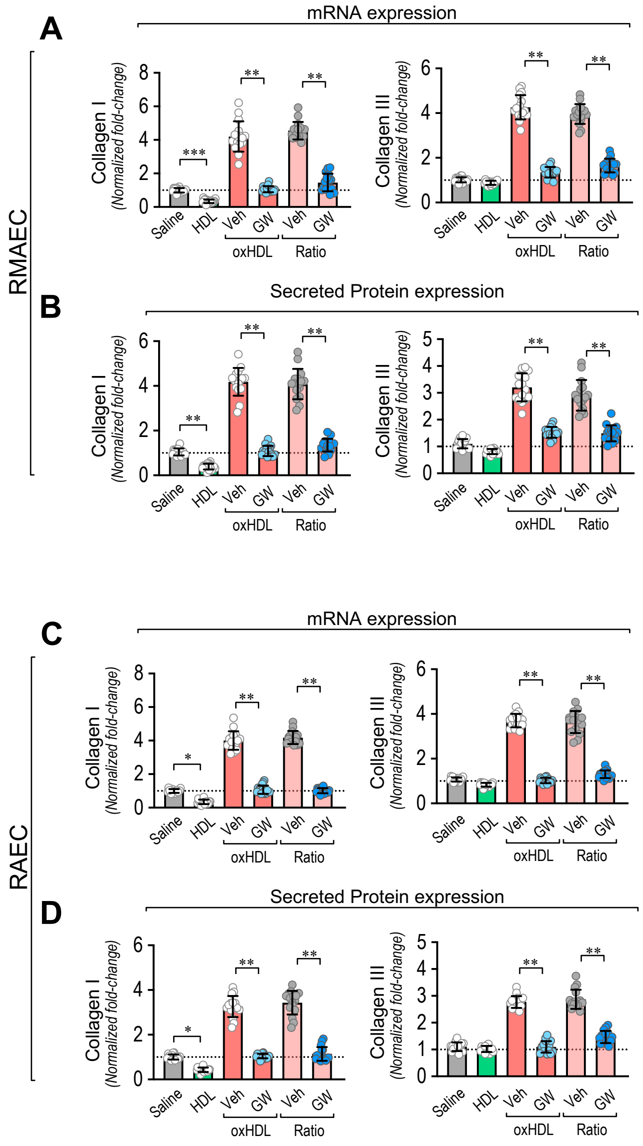

Supplemental Figure S9

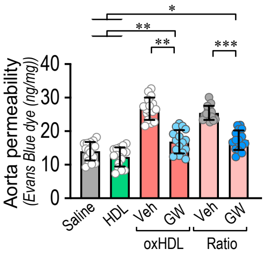

Supplemental Figure S10

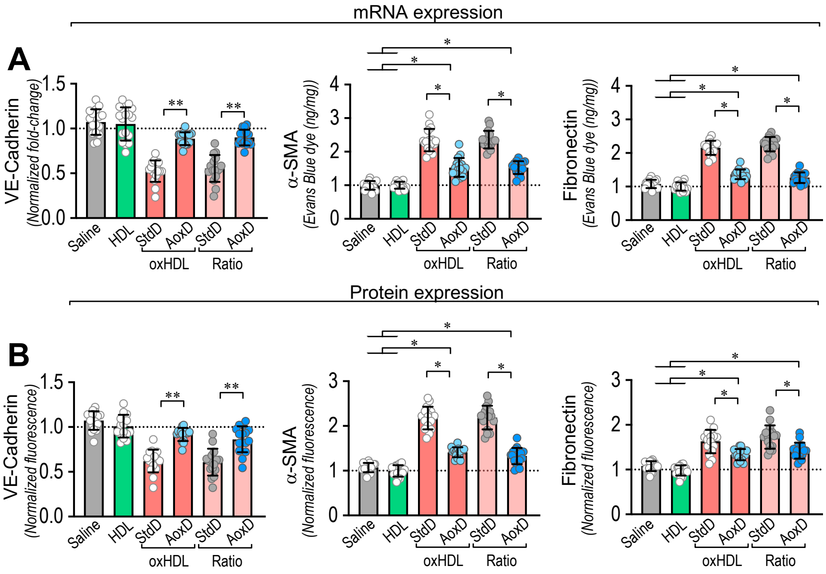

Supplemental Figure S11

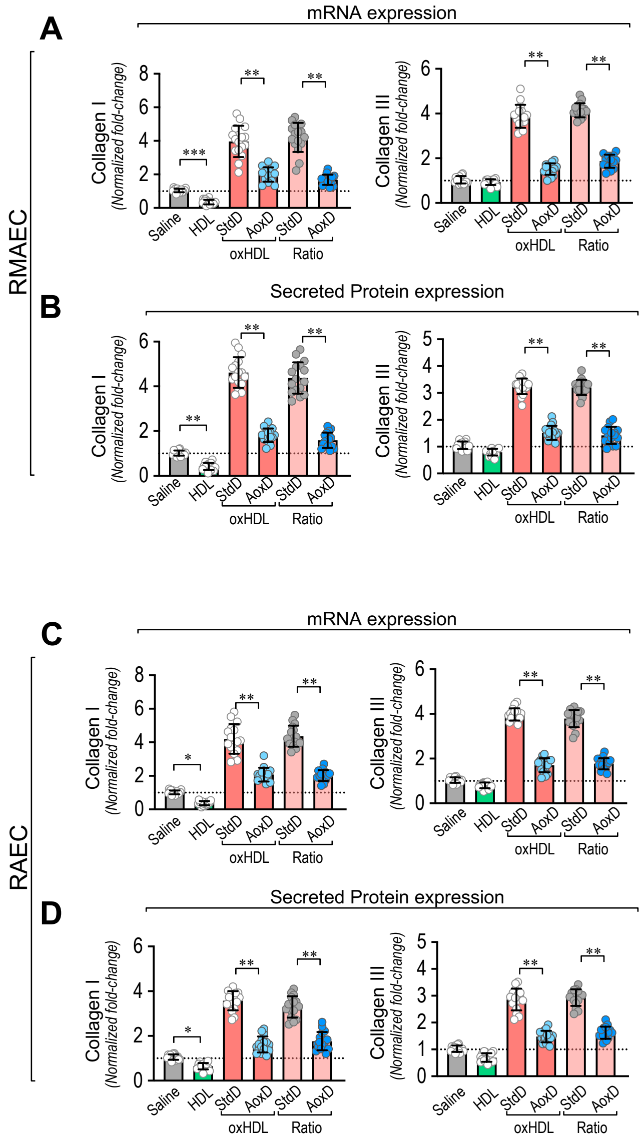

Supplemental Figure S12

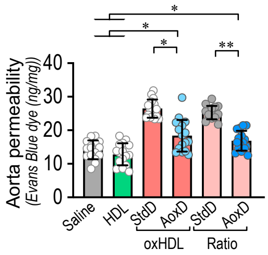

Supplemental Figure S13

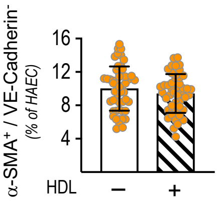

Supplemental Figure S14

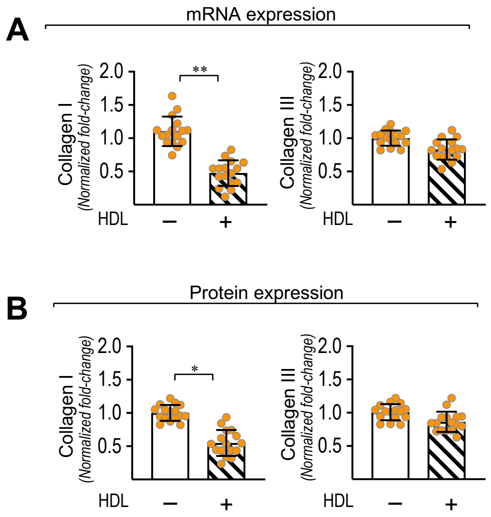

Supplement: Supplementary file 1 [file antioxidants-11-02469-s001.zip › antioxidants-2048340-supplementary.pdf]
